# Polydopamine-based Nanoadjuvants Promote a Positive Feedback Loop for Cancer Immunotherapy via Overcoming Photothermally Boosted T Cell Exhaustion

**DOI:** 10.34133/bmr.0166

**Published:** 2025-03-19

**Authors:** Xiao-Kai Chi, Hai-Rui Zhang, Jing-Jing Gao, Jin Su, Yong-Zhong Du, Xiao-Ling Xu

**Affiliations:** ^1^ Shulan International Medical College, Zhejiang Shuren University, Hangzhou 310015, PR China.; ^2^ College of Pharmacy, Jiamusi University, Jiamusi 154007, PR China.; ^3^ Institute of Pharmaceutics, College of Pharmaceutical Sciences, Zhejiang University, Hangzhou 310058, PR China.

## Abstract

Immunogenic cell death, triggered by photothermal therapy or specific chemotherapy, strives to establish a positive feedback loop in cancer immunotherapy. This loop is characterized by the rapid release of antigens and adenosine triphosphate (ATP), ultimately leading to accelerated T cell infiltration. However, this loop is hindered by T cell exhaustion caused by adenosine originating from ATP and glucose deprivation in the immunosuppressive microenvironment. To overcome this challenge, we developed a pH-low insertion peptide-functionalized mesoporous-polydopamine-based nanoadjuvant that incorporates adenosine deaminase and doxorubicin (termed as PPMAD). PPMAD aimed to overcome T cell exhaustion by reducing adenosine consumption and providing an alternative carbon source for CD8^+^ T cell function during glucose starvation. First, PPMAD triggered the burst release of antigens and ATP through photothermal therapy and doxorubicin-induced immunogenic cell death, culminating in the expedited infiltration of T cells. Second, adenosine deaminase depleted adenosine, reducing immunosuppressive agents and generating abundant inosine, which served as an alternative carbon source for CD8^+^ T cells. By implementing this “reducing suppression and broadening sources” strategy, we successfully overcome T cell exhaustion, greatly enhancing the effectiveness of cancer immunotherapy both in vitro and in vivo. Our findings highlighted the positive feedback loop between on-demand photothermal therapy, chemotherapy immunotherapy, and achieving complete tumor response.

## Introduction

Malignant breast cancers pose a significant challenge for effective treatment, particularly highly metastatic triple-negative breast cancers that lack therapeutic targets such as estrogen receptor, progesterone receptor, and human epidermal growth factor receptor 2 molecules [1–4]. However, in recent years, cancer immunotherapy has emerged as a promising approach to enhancing antitumor responses by leveraging the body’s immune system. This novel and durable treatment option has shown efficacy in certain types of cancer characterized by immune cell infiltration [5–8].

For instance, researchers have employed photothermal therapy or specific chemotherapy to induce immunogenic cell death (ICD), aiming to establish a positive feedback loop within cancer immunotherapy. This process involves the rapid release of damage-associated molecular patterns and adenosine (ADO) triphosphate (ATP), which leads to the maturation of dendritic cells (DCs) and subsequent activation of T cells, thereby promoting tumor-specific cellular immunity [9–12].

However, this feedback loop has certain limitations. First, the abundant ATP released during ICD can be metabolized to ADO, a potent immunosuppressor, by enzymes such as CD39 and CD73. The engagement of ADO with ADO 2A receptors (A2AR), which act as immune checkpoints on various immune cell surfaces, ultimately induces exhausted antigen-presenting cells and T cells, resulting in suboptimal cancer immunotherapy [13–15]. Second, highly glycolytic cancer cells may deplete nutrients in the tumor microenvironment, particularly glucose, thereby limiting glucose availability to T cells. Previous studies have shown that activated murine T cells significantly enhance glucose catabolism, and glucose starvation compromises the viability and proliferation of effector T cells, ultimately leading to accelerated T cell exhaustion. Therefore, the paradoxes between ICD-induced antitumor immunity and T cell exhaustion remain a formidable challenge [16–18].

Fortunately, several strategies have emerged to target ADO for immunomodulation, thereby alleviating ICD-based T cell exhaustion in vivo. For example, Jadidi-Niaragh et al. [19] synthesized chitosan-lactate nanoparticles loaded with CD73-specific small interfering RNA, which effectively suppressed CD73 expression in tumor cells in vivo. This elicited a heightened antitumor immune response, restrained the infiltration of immunosuppressive cells within the tumor microenvironment, impeded tumor growth, and prevented metastasis. Qi et al. [20] developed E-selectin–modified thermosensitive micelles (ES-DSMs) by coloading doxorubicin (DOX) with an A2AR inhibitor. ES-DSMs induced tumor ICD through the rapid release of DOX, eliciting tumor-specific immunity. The inclusion of the A2AR inhibitor alleviated immunosuppression caused by the ADO pathway, further enhancing the ICD-based antitumor immune response. However, neither approach fundamentally addresses the issue of insufficient energy supply to T cells, thus failing to fully reverse T cell exhaustion. Therefore, it is still necessary to explore new approaches that not only reduce immunosuppressive substances within the tumor microenvironment but also provide a continuous and alternative energy source to sustain the vitality of T cells, enabling long-lasting tumor immunotherapy.

The emergence of ADO deaminase (ADA) provides a new approach to reshaping the microenvironment. ADA is an enzyme involved in purine metabolism that catalyzes the removal of ammonia from ADO, generating inosine. The produced ammonia can alkalize the lactate within the microenvironment. In addition, inosine serves as an alternative energy source for antitumor effector T cells and can restore the proliferation and cytotoxicity of effector T cells under conditions of glucose deprivation. It acts as an important immunostimulant that mediates immune responses [21–23].

**Fig. 1.**
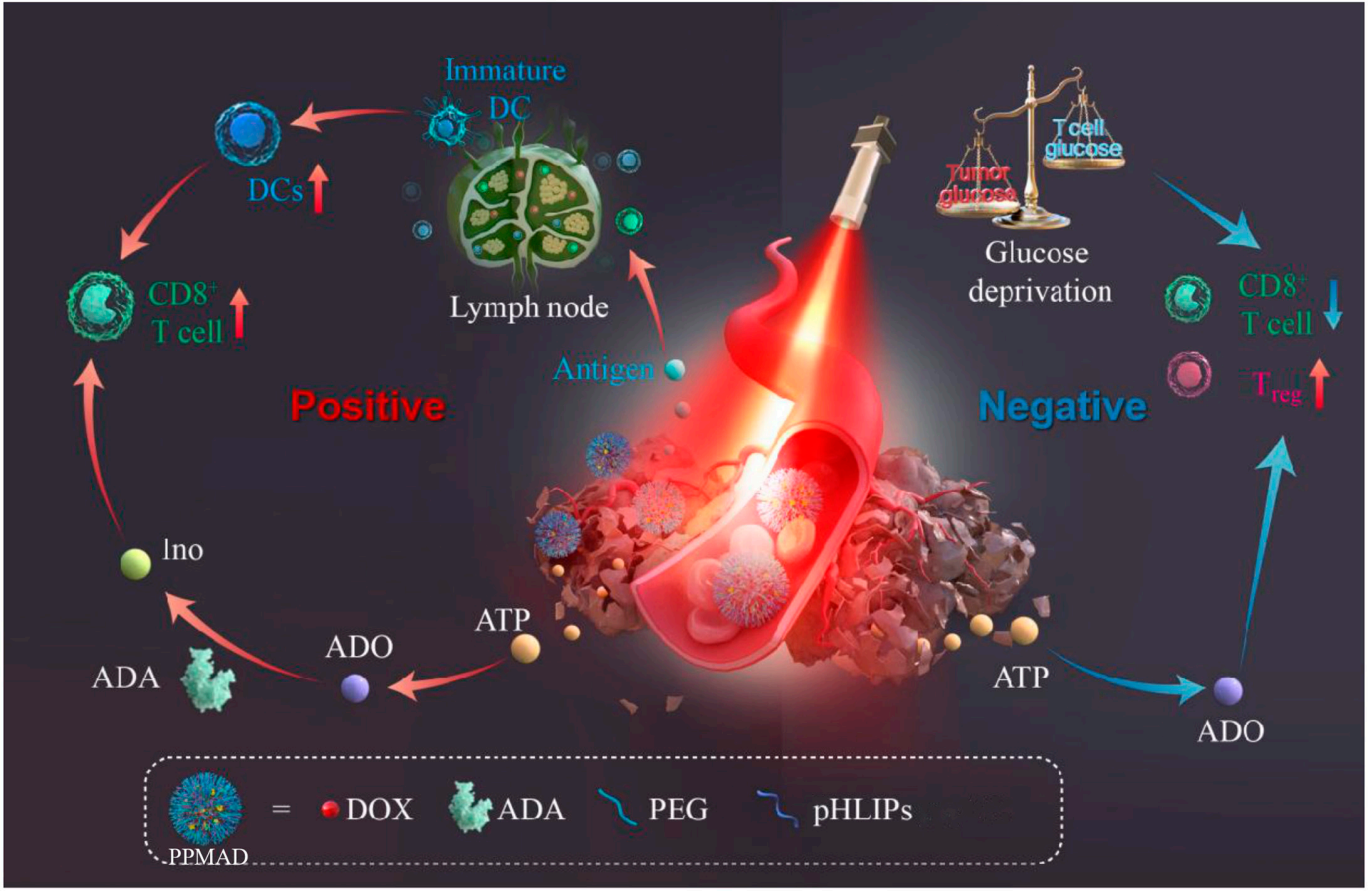
Schematic illustration of PPMAD for photodynamic activated immunotherapy against metastatic tumors. Ino, inosine.

Hence, we developed PPMAD, a pH-low insertion peptide (pHLIP)-functionalized mesoporous-polydopamine (MPDA)-based nanoadjuvant that incorporated ADA and DOX. PPMAD was designed as a novel nanoadjuvant to address T cell exhaustion by reducing ADO consumption and providing an alternative carbon source for CD8^+^ T cell function during glucose deprivation. It facilitated the rapid release of antigens and ATP through photothermal therapy and DOX-induced ICD, resulting in enhanced T cell infiltration at the tumor site. In addition, ADA depleted ADO, reducing immunosuppressive agents and increasing inosine levels, which served as an alternative carbon source for CD8^+^ T cells. This “reducing suppression and broadening sources” strategy underscored the potential positive feedback loop between on-demand photothermal therapy, chemotherapy immunotherapy, and the achievement of a comprehensive and favorable tumor response (Fig. 1).

## Materials and Methods

### Materials and animals

Dopamine hydrochloride and ammonia (NH_3_·H_2_O, 25% to 28%) were purchased from Aladdin Bio-Chem Technology Co. Ltd. (Shanghai, China). Pluronic F-127, poly(ethylene glycol)-block-poly(propylene glycol)-block-poly(ethylene glycol) (Pluronic P-123), and 1,3,5-trimethylbenzene (TMB) were purchased from Sigma-Aldrich (St. Louis, MO). NH_2_-polyethylene glycol (PEG)-NH_2_ was purchased from Toyongbio Co. Ltd. (Shanghai, China). pHLIP-N3 [series: K(N3)GGACDDQNPWRAYLDLLFPTDTLLLDLLWKK] was purchased from DGpeptides Co. Ltd (Wuhan, China). Cell Counting Kit-8 (CCK8) was purchased from Dalian Meilun Biotechnology Co. Ltd. (Dalian, China). ADA was purchased from Shanghai Yuanye Bio-Technology Co. Ltd. (Shanghai, China). DOX hydrochloride was purchased from Aladdin Bio-Chem Technology Co. Ltd. (Shanghai, China). Enzyme-linked immunosorbent assay (ELISA) ADO assay kit was purchased from mmbio Co. Ltd. (Shanghai, China). All other chemicals used in the current work were of analytical or chromatographic grades.

BALB/c mice aged 6 to 8 weeks and weighing 20 g were provided by Shanghai Silaike Laboratory Animal Limited Liability Company. They were offered sufficient food and water. All animal experiments were performed on the basis of the National Institutes of Health (USA) guidelines for the care and use of laboratory animals in research. Moreover, the surgical procedures during the experiments were approved by the Committee for Animal Experiments of Zhejiang University (26978).

### Synthesis and characterization of pHLIP-modified PEG-MPDA nanoparticles

Here, the MPDA nanoparticles were prepared using the soft template method [24]. Briefly, 0.15 g of dopamine hydrochloride, 0.075 g of Pluronic F-127, and 0.03 g of Pluronic P-123 were precisely weighed. They were then mixed with 20 ml of 40% ethanol and sonicated in a water bath until a clear and transparent solution was formed. Next, 0.4 ml of TMB was added, and the solution was sonicated for 5 min (600 W, working for 3 s and paused for 2 s) until it changed from a colorless transparent solution to a milky white liquid. After that, 0.4 ml of ammonia was added, and the solution was stirred at room temperature for 4 h (800 rpm) to allow dopamine to slowly oxidize and aggregate. The mixture was then centrifuged (9,500 rpm for 15 min), and the polydopamine nanoparticles were collected. They were washed with ethanol to remove impurities. The obtained MPDA nanoparticles were dispersed in pure water, and NH_2_-PEG-NH_2_ was added (*m*_MPDA_:*m*_NH2-PEG-NH2_ = 1:2) to construct PEG-modified dopamine nanoparticles.

pHLIP-modified PEG-modified copolymer was synthesized as follows: 5 mg of pHLIP-N3 was dissolved in 50 μl of dimethyl sulfoxide and placed in 1 ml of pure water. Then, 7 mg of dibenzocycloocyne (DBCO)-PEG-NH_2_ (*n*_pHLIP-N3_:*n*_DBCO-PEG-NH2_ = 1:1) was added to the system. The solution was continuously stirred overnight, followed by dialysis [molecular weight cutoff (MWCO) = 3,000 to 5,000 Da] for 12 h with water changed every 4 h. The resulting graft was freeze-dried, and the structure of pHLIP-PEG-NH_2_ was confirmed by ^1^H-nuclear magnetic resonance (NMR).

Six milligrams of PEG-MPDA (PM) was mixed with 12 mg of pHLIP-PEG-NH_2_ (*m*_PM_:*m*_pHLIP-PEG-NH2_ = 1:2). The mixture was stirred overnight and then centrifuged to obtain pHLIP-PM (PPM; 9,500 rpm for 15 min). The particle size of PM and PPM was analyzed using dynamic light scattering, and the morphological examination was performed using transmission electron microscopy (TEM).

To explore the photothermal conversion efficiency, the PM was irradiated with near-infrared (NIR) light at 808 nm, and the temperature was monitored by a digital thermometer every 30 s:

$$\eta =\frac{m_{s}C_{s}\left(T_{\max}-T_{\text{surr}}\right)-Q_{\mathrm{dis}}}{\tau I\left(1-10^{-A_{808}}\;\right)}$$ (1)

where $T_{\max}$ is the equilibrium temperature, $T_{\text{surr}}$ is the ambient temperature of the surroundings, $Q_{\mathrm{dis}}$ is the heat associated with the light absorbance by the solvent, I is the incident laser power, $A_{808}$ is the absorbance of the PPM at 808 nm, ζ is the sample system time constant, $m_{s}$ is the mass of water used as the solvent, $C_{s}$ is the heat capacity of water used as the solvent [25].

### Preparation of polydopamine nanoparticles loaded with DOX and ADA

The previously prepared PPM was sonicated for 5 min (250 W, working for 3 s and stopping for 2 s). The dried and weighed PPM was then reconstituted to a volume of solution (5 mg/ml). From this solution, 1 ml was taken, and 50 U of ADA was added and stirred for 6 h. After that, 700 μg of DOX was added and stirred overnight in the dark. The solution was then centrifuged (9,500 rpm/min for 15 min), and pure water was added for resuspending and washing until the volume reached 1 ml.

The loading of ADA was detected using the ADA kit, and the loading of DOX was measured by fluorescence spectrophotometry using a microplate reader at 470 to 580 nm. The encapsulation efficiency (EE) and drug loading (DL) were calculated using the following formulas:

$$\begin{array}{c} \mathrm{DL}\;\left(\%\right)=\left(\text{mass of}\;\mathrm{DOX}\;\text{encapsulated in MPDA}/\right.\\ \left.\text{mass of}\;\mathrm{DOX}-\text{loaded MPDA}\right)\times 100\% \end{array}$$ (2)

$$\begin{array}{c} \mathrm{EE}\;\left(\%\right)=\left(\text{mass of}\;\mathrm{DOX}\;\text{encapsulated in MPDA}/\right.\\ \left(\text{mass of}\;\mathrm{DOX}\;\text{added}\right.\times 100\% \end{array}$$ (3)

### In vitro release of DOX

Dialysis membrane diffusion technology was used to assess the drug release characteristics. Phosphate buffers with different pH gradients were prepared to investigate the release behavior of DOX. A known amount of PPMAD nanoparticle solution was sealed in a dialysis membrane (MWCO = 14,000 Da) and immersed into 50 ml of release medium. The experiment was conducted in a constant temperature shaker maintained at 37°C and shaken horizontally at 60 rpm. At predetermined time points, the release medium was removed, and the same volume of fresh release medium was added. The released DOX was measured by fluorescence spectrophotometry.

### In vitro photothermal experiments

To evaluate the photothermal properties of PPMAD, the photothermal effect of PPMAD was investigated at different concentrations with the same power density or at different power densities with the same concentration. The temperature of the solution was recorded using a digital thermometer, and the change in temperature was captured using infrared thermography at specific time intervals. PPMAD was placed in a 96-well plate and irradiated with 808-nm NIR light for 300 s at various power densities. The temperature of 1 sample was recorded every 30 s using a digital thermometer. The photothermal stability of PPMAD was assessed by subjecting the sample to 3 cycles of irradiation for 300 s, followed by natural cooling for 300 s [26–28].

### Cell culture

For cell culture, mouse 4T1 breast cancer cells (serial number: TCM32) were obtained from the Cell Bank of the Chinese Academy of Sciences (Shanghai, China). The 4T1 cells were cultured in a modified RPMI 1640 supplemented with 10% fetal bovine serum and 1% penicillin-streptomycin at a temperature of 37 °C and a 5% carbon dioxide atmosphere.

### Biosafety and in vitro cytotoxicity

The biosafety of the vector was assessed using the CCK8 method. One hundred microliters of 4T1 cells was seeded into each well of a 96-well plate at a density of 5 × 10^3^ cells per well. After 24 h, different concentrations of PPM were added to the plates. After 4 h, the cells were subjected to laser treatment (808-nm NIR light irradiated at 1.5 W/cm^2^ for 8 min) or no laser treatment. The plates were then incubated for an additional 24 h, followed by the addition of 10 μl of CCK8 solution to each well. After incubating for 2 h, the absorbance at a wavelength of 450 nm was recorded using a microplate reader.

To evaluate the cytotoxicity of PPMAD on 4T1 cells, the CCK8 method was employed. One hundred microliters of 4T1 cells was seeded into each well of a 96-well plate at a density of 5 × 10^3^ cells per well. After 24 h of incubation, Free DOX, PPM, PPMA, and PPMAD were added to the cells, followed by a 4-h incubation period. The cells were then subjected to laser treatment (808-nm NIR light irradiated at 1.5 W/cm^2^ for 8 min) or no laser treatment. After an additional 24 h of incubation, 10 μl of CCK8 solution was added to each well, and the plates were incubated for 2 h. The absorbance at a wavelength of 450 nm was recorded using a microplate reader.

### Cell uptake and staining of live dead cells

To assess the uptake capacity of 4T1 cells for PPMAD, fluorescence microscopy was used. 4T1 cells were seeded in six-well plates at a density of 5 × 10^5^ cells per well and cultured for 24 h. PMAD and PPMAD were then added to the cells and incubated for 2, 4, and 6 h. The supernatant was discarded, and the cells were rinsed with phosphate-buffered saline (PBS) buffer, fixed with paraformaldehyde, and stained with 4tained witino-2-phenylindole (DAPI) dye. The cells were observed using fluorescence microscopy, and the fluorescence intensity was quantified using ImageJ software.

For the investigation of cell viability, 4T1 cells were seeded in a 96-well plate at a density of 1 × 10^4^ cells per well and incubated for 24 h. PPMAD was added to the cells, followed by a 4-h incubation period. The cells were then subjected to laser treatment (808-nm NIR light irradiated at 1.5 W/cm^2^ power for 8 min) or no laser treatment. Acetoxymethyl ester (AM)/propidium iodide (PI) dye was added to investigate cell viability. The cells were washed with PBS after dye treatment, and a fluorescence microscope was used for observation.

### Maturation of bone-marrow-derived DCs

To induce the maturation of bone-marrow-derived DCs (BMDCs) using PPMAD, BMDCs were extracted from bone marrow precursors from Balb/c mice. 4T1 cells were seeded into a 96-well plate at a density of 5 × 10^3^ cells per well and incubated overnight. PPMAD was added to the cells, followed by a 4-h incubation period. The cells were then irradiated with 808-nm NIR light at a power density of 1.5 W/cm^2^ for 8 min. BMDCs were added for coincubation, and after 24 h, the cells were digested. Flow cytometry fluorescent antibodies were added for staining, and the relevant immune factors were analyzed using flow cytometry.

### Lymphocytic T cell differentiation and maturation

To induce lymphocytic T cell differentiation and maturation using PPMAD, 4T1 cells were seeded into a 96-well plate at a density of 5 × 10^3^ cells per well and incubated overnight. PPMAD was added to the cells, followed by a 4-h incubation period. The cells were then irradiated with 808-nm NIR light at a power density of 1.5 W/cm^2^ for 8 min. Lymphoid T cells and BMDCs were added, and the cells were coincubated for 24 h. The cells were digested, and flow cytometry fluorescent antibodies were added for staining. After staining, PBS was washed 3 times, and flow cytometry was used to analyze the relevant immune factors. The cell coincubation supernatant was collected and measured using the ELISA ADO assay kit (method according to instructions).

### In vivo distribution

An orthotopic 4T1 tumor model was established by subcutaneously injecting 4T1 cells at a density of 1 × 10^6^ cells per mouse breast pad. Once the tumor volume reached 100 mm^3^, the mice were intravenously injected with free indocyanine green (ICG), ICG-PPM, and ICG-PM. At 2, 12, 24, and 48 h after administration, the distribution of different preparations in 4T1-tumor-bearing mice was observed using the In Vivo Imaging System (IVIS) spectroscopy system. After 48 h, the mice were euthanized, and their main organs, including the heart, liver, spleen, lung, kidney, and tumors, were collected. The IVIS spectroscopy system was used to detect fluorescence signals in these organs.

### In vivo therapeutic effect

A total of 1 × 10^6^ 4T1 cells were subcutaneously injected into the breast pads of mice. When the tumor volume reached 50 to 100 mm^3^, the tumor-bearing mice were divided into 8 groups: PBS, PBS(+), free DOX, free DOX(+), PPMA(+), PPMD(+), PMAD(+), and PPMAD(+). The symbol (+) represents laser treatment, where 808-nm NIR light was irradiated at a power of 1W/cm^2^ for 3 min. Each mouse was dosed at intervals, and equivalent doses of the drug were administered on days 1, 3, and 5, totaling 3 times. Laser treatment at 1 W/cm^2^ was given on days 2, 4, and 6, with each laser irradiation lasting 3 min. The tumor volume of each mouse was monitored with vernier calipers every other day, and tumor diameter data were recorded daily from the first day until the end of the treatment cycle. Tumor volume was defined as *A*^2^ × *B* / 2. In addition, the body weight of each mouse was recorded every other day. After the completion of treatment on the 15th day, all mice were euthanized, and their blood and main organs were collected. The main organs (heart, liver, spleen, lung, kidney, and tumor) were fixed in 4% paraformaldehyde, embedded in paraffin, cut into 5-μm slices, and stained with hematoxylin and eosin (H&E). To demonstrate the ICD effect of tumor tissues, the exposure level of calreticulin (CRT) was investigated using immunofluorescence and laser confocal scanning microscopy. The infiltration of CD8^+^ T cells and CD44^+^ cells in the spleen was analyzed using immunofluorescence, and the apoptosis of tumor cells was observed through immunohistochemical study using Ki67 and terminal-deoxynucleotidyl-transferase-mediated deoxyuridine triphosphate nick end labeling (TUNEL) staining. The routine blood status of mice was analyzed using the Beckman automatic analyzer.

### Immunity level

T cells in organs were isolated using density gradient centrifugation. The spleen of mice was taken, and the tumors were filtered and ground with a 70-μm strainer. Red blood cells were washed multiple times with PBS and then lysed by centrifugation. After resuspension, the red blood cells were washed 3 times with PBS buffer to obtain the cell suspension. CD3, CD4, and CD8 flow cytometry fluorescent antibodies were added for staining and labeling. After incubating in the dark at room temperature for 30 min, the PBS buffer was washed 3 times. Flow cytometry was used to investigate the relevant immune factors. The tumor was harvested, and an appropriate volume of PBS was added to create a tumor tissue slurry using a homogenizer. This mixture was then centrifuged at 1,000 rpm for 5 min to collect the tumor supernatant. The ADO concentration in the supernatant was subsequently analyzed using an ELISA ADO detection kit, following the manufacturer’s instructions.

### Biochemical monitoring and biosafety

Plasma was collected to measure the levels of alanine aminotransferase (ALT) and creatinine to investigate liver and kidney function and evaluate biological safety.

### Statistical analysis

Comparative analyses of differences between groups were calculated using Prism 7.0 with a 95% confidence interval, using 1-way analysis of variance (ANOVA). Significant differences were denoted as ***P < 0.001, **P < 0.01, and *P < 0.05. Values are presented as means ± SD.

## Results

### Preparation and characterization of PPMAD nanoadjuvants

**Fig. 2.**
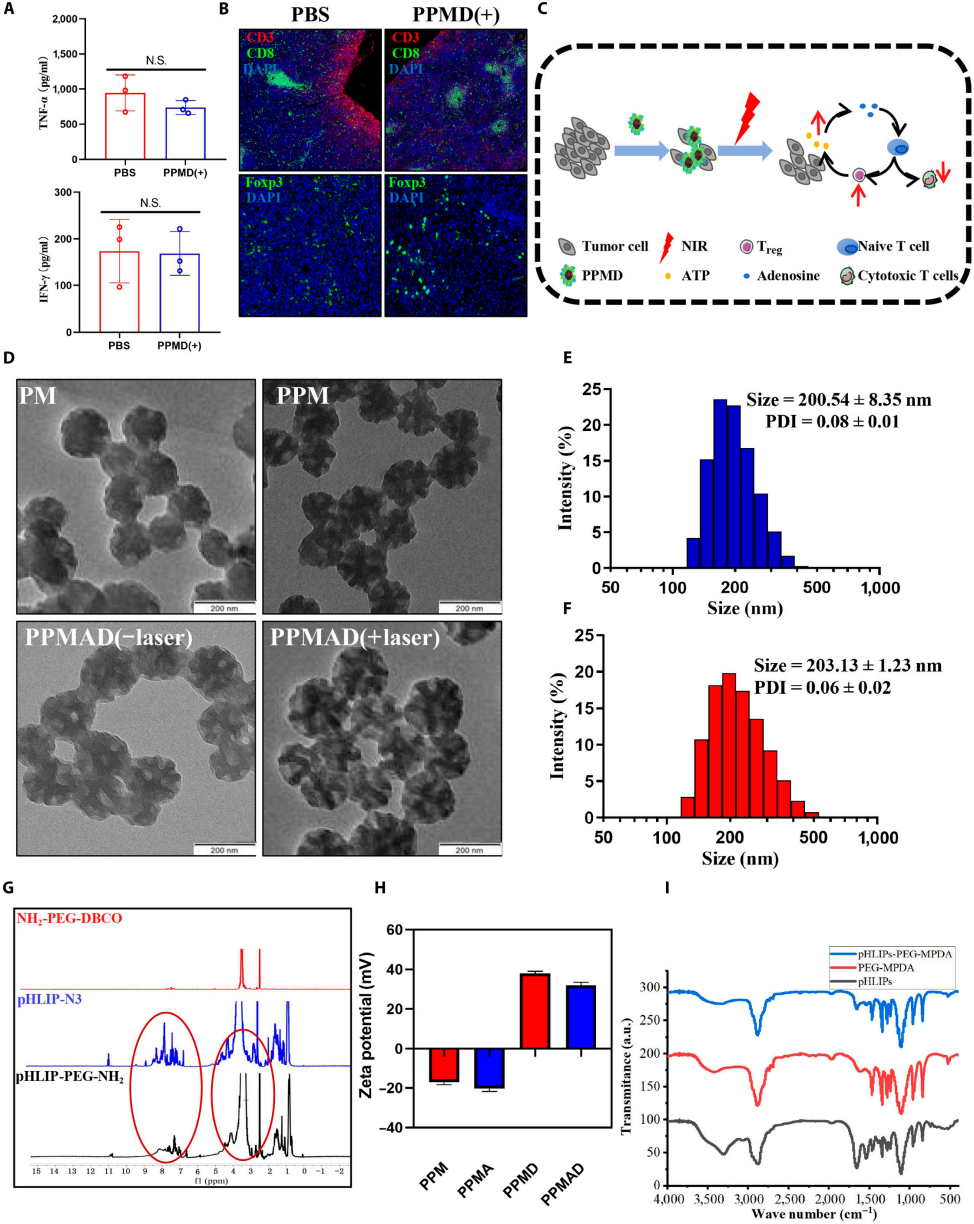
(A) Interferon-γ (IFN-γ) and tumor necrosis factor-α (TNF-α) levels in mouse tumors after PBS treatment or PPMD(+) treatment. N.S., not significant. (B) CD3^+^CD8^+^ expression levels in the spleen of mice and Foxp3 in tumors after PBS treatment or PPMD(+) treatment. (C) Schematic diagram of the intensification of local immunosuppression in tumors triggered by the ICD effect. (D) TEM images of PM, PPM, PPM-ADA-DOX, and PPM-ADA-DOX after irradiation (1.5 W/cm^2^ for 300 s). Scale bars, 200 nm. (E) The particle size distribution of PPM and (F) PPMAD. (G) ^1^H NMR of NH_2_-DBCO, pHLIP-N_3_, and pHLIP-NH_2_. (H) Zeta potential of PPM and PPMAD. (I) Fourier transform infrared spectra of pHLIP, PM, and PPM. a.u., arbitrary units.

According to our previous study, photothermal therapy combined with chemotherapy drugs for antitumor therapy was not able to improve the immunosuppressive phenomenon of the tumor microenvironment (Fig. 2A and B). In addition, the ICD effect generated during treatment can also exacerbate the degree of local immunosuppression of tumors (Fig. 2C). On the basis of previous studies, MPDA nanoparticles were initially prepared using the soft template method, as depicted in Fig. S1. Since pHLIP is a peptide, its primary structure affects its properties and functions and significantly influences them. In our analysis, we focus on the key functional groups of pHLIP-N3 to elucidate the construction of polymer. Subsequently, pHLIP-PEG-NH_2_ was synthesized through a copper-free click reaction between NH_2_-PEG-DBCO and pHLIP-N3, which is the azide group of the pHLIP-terminus-targeting peptide for tumor cells. The successful synthesis of pHLIP-PEG-NH_2_ was confirmed by ^1^H NMR, showing characteristic peaks at 3.5 and 7.5 parts per million (ppm) for NH_2_-PEG-NH_2_ (–O–CH_2_CH_2_–) and pHLIP(–NH_2_), respectively. The chemical shift at 11 ppm corresponds to the –COOH group, while the shift at 1.8 ppm is associated with the CH3-CH2 group (Fig. 2G). In Fig. 2I, the samples are arranged from top to bottom as follows: PPM, PM, and pHLIP. The peak at 1,655 cm^−1^ arises from the overlap of the resonant vibrations of C=C in the aromatic ring and the bending of N–H in the dopamine structure. The peaks at 1,286 and 1360 cm^−1^ are attributed to the stretching and bending vibrations of C–O–H on the benzene ring, respectively. The peak at 3,319 cm^−1^ represents the stretching vibration absorption of the C–N bond. The characteristics of the MPDA peak indicate successful polymerization, as evidenced by the significant increase in peak intensity at 1,530 cm^−1^ for PPM, which results from the introduction of additional N–H groups on the surface of MPDA. This suggests that pHLIPs have been successfully constructed with MPDA [29]. Compared to unmodified MPDA, the dispersibility of MPDA in different solvents was significantly improved after pHLIP-PEG-NH_2_ modification (Fig. S2). Particle size measurements revealed that PPM had a hydrated particle size of approximately 200.54 ± 8.35 nm, a polymer dispersity index (PDI) of about 0.06 ± 0.02, and exhibited uniform size distribution. TEM images showed that PPM exhibited a spherical or spherical-like shape with a distinct mesoporous surface structure and the particle size was approximately 200 nm (Fig. 2D). The 200-nm nanoparticles meet our application needs. (a) In vivo distribution: Nanoparticles in the size range of 100 to 300 nm exhibit optimal tumor accumulation due to the enhanced permeability and retention effect. Particles smaller than 100 nm tend to be rapidly cleared from circulation, resulting in reduced accumulation in vivo. Conversely, particles larger than 300 nm face challenges in passing through the gaps between endothelial cells, which can hinder their distribution and pose safety risks due to prolonged retention. (b) Photothermal conversion efficiency: Smaller nanoparticles generally demonstrate higher photothermal conversion efficiency. While this property can enhance ICD, it is important to note that reduced particle size may also lead to accelerated ATP release and increased T cell exhaustion.

To achieve a balance between biodistribution, photothermal conversion efficiency, and T cell exhaustion, we concluded that a particle size of 200 nm is optimal.

Next, PPMAD nanoparticles loaded with ADA and DOX were prepared using a previously established method. The particle size analysis indicated that PPMAD had a hydrated particle size of around 203.13 ± 1.23 nm, with a PDI of about 0.08 ± 0.01, demonstrating uniform size distribution. The TEM images confirmed that the drug-loaded nanoparticles maintained a spherical or spherical-like shape, with a particle size similar to that of PPM (Fig. 2E and F). The zeta potential changed from −17.17 ± 2.35 to 31.96 ± 1.97 mV after DL (Fig. 2H). The DOX loading was determined to be 7.52 ± 0.41%, with an EE of 64.35 ± 3.15% using fluorescence spectrophotometry. The loading of ADA was determined to be 10.58 ± 0.51%, with an EE of 72.78 ± 3.40%. We further assessed the activity of ADA using Berthelot colorimetry and found that ADA retained high activity following dopamine loading. When evaluated using the ADA kit, the concentration of ADA was measured at 28 ± 0.57 U/ml.

**Fig. 3.**
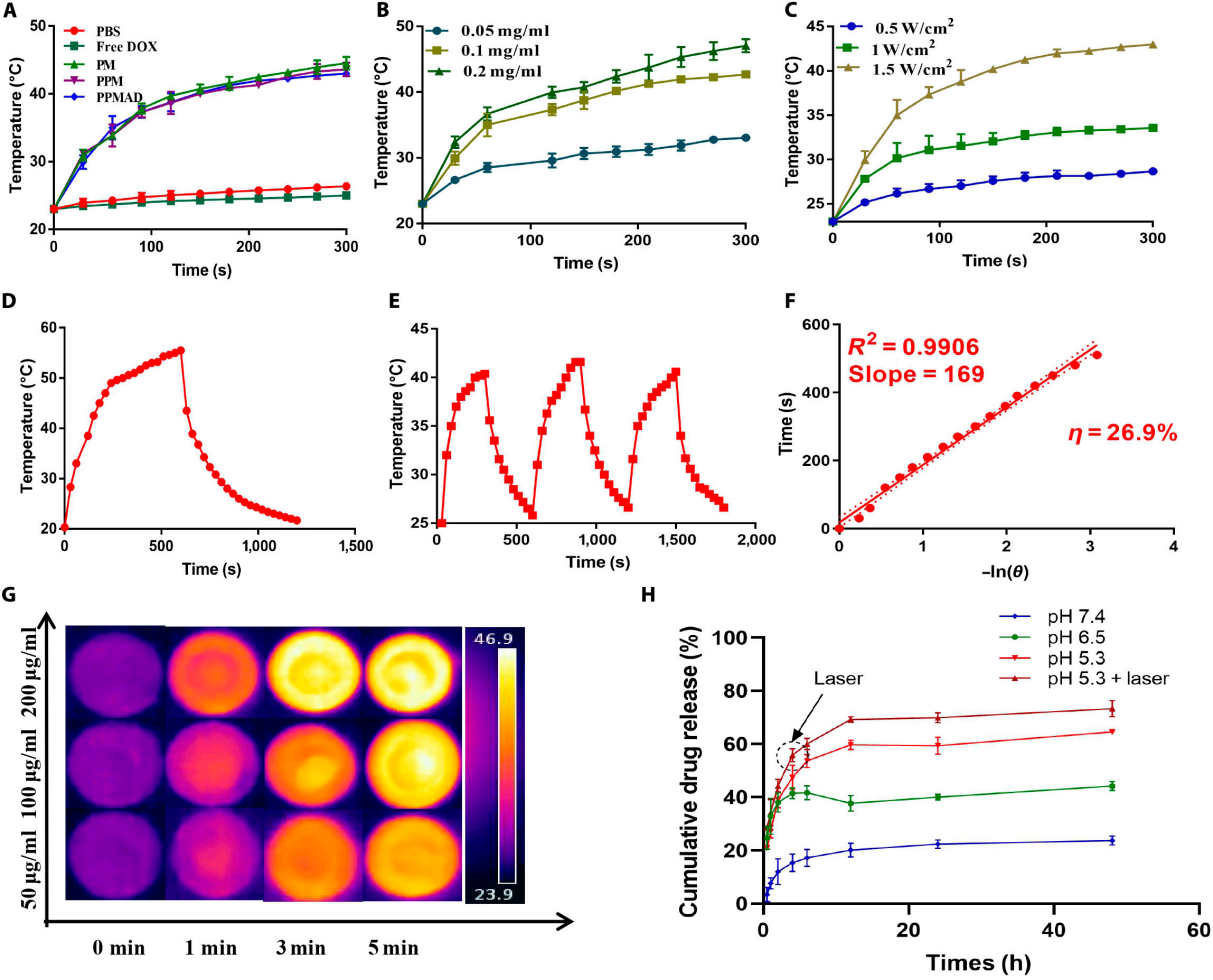
Photothermal conversion of PPMAD under irradiation of the NIR laser. (A) The changes in the fluorescent intensity of different preparations (100 μg/ml) under NIR irradiation (1.5 W/cm^2^ for 300 s). (B) Temperature elevation of PPMAD at various concentrations (808 nm at 1 W/cm^2^). (C) The changes in the temperature of PPMAD (100 μg/ml) with the irradiation by the 808-nm NIR light at a different power density of 0.5, 1.0, and 1.5 W/cm^2^ for 300 s. (D) Photothermal conversion of PPMAD under irradiation of the NIR laser (808 nm at 1 W/cm^2^). (E) Temperature changes of PPMAD (MPDA concentration, 100 μg/ml) against irradiation time of 808-nm NIR laser. (F) Linear time date versus −ln(*θ*) obtained from the cooling period. (G) Infrared thermal images of different concentrations of PPMAD, laser-irradiated (808 nm at 1.5 W/cm^2^). (H) In vitro cumulative release of DOX from PPMAD (*n* = 3).

Subsequently, the photothermal conversion performance of PPMAD was studied by investigating the temperature change under NIR irradiation at different powers (0.5 to 1.5W/cm^2^) for 300 s. As shown in Fig. 3A, this confirms the photothermal conversion ability of PPMAD. PPMAD exhibited a significant temperature increase compared to the free DOX and PBS groups, indicating its ability to generate heat upon NIR light irradiation. Moreover, there was no significant difference compared to the PPM group, suggesting that the DL did not affect the conversion of light energy to heat energy. Further analysis revealed that the concentration of PPMAD and the power of NIR light influenced the level of thermal energy (Fig. 3B, C, and G). The photothermal stability of PPMAD was also investigated, demonstrating good stability and retention of photothermal conversion ability over 3 heating and cooling cycles (Fig. 3D and E). The photothermal conversion efficiency of PPMAD was calculated to be 26.9% (Fig. 3F), indicating excellent photothermal conversion performance.

To simulate the drug release behavior in the tumor microenvironment, the in vitro release of PPMAD was investigated under low pH conditions and NIR light intervention. As shown in Fig. 3H, PPMAD exhibited minimal release at pH 7.4, but the release ability significantly improved under conditions mimicking the tumor microenvironment. With NIR light irradiation, the drug release was further enhanced, reaching over 70% under pH 5.3 with laser irradiation. Overall, these results demonstrate that PPMAD possesses excellent photothermal conversion performance, good stability, pH-responsive behavior, and responsiveness to NIR light stimulation.

### In vitro cytotoxicity

**Fig. 4.**
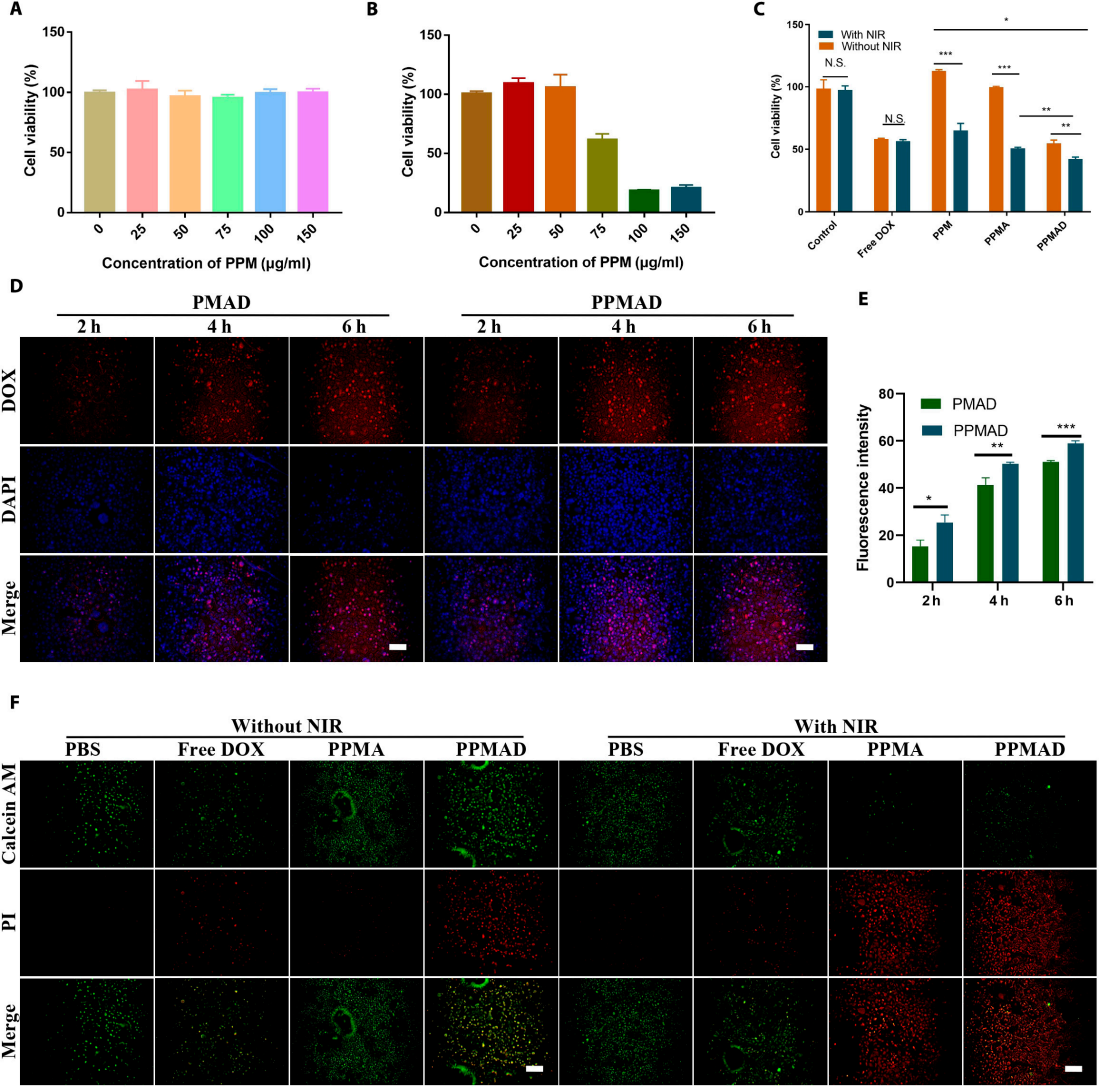
The cytotoxicity of PPMAD and its cellular uptake behavior. (A) The cell viability of PPMAD without laser irradiation toward 4T1 cells. (B) The cell viability of PPMAD with laser irradiation toward 4T1 cells. (C) The cell viability of different preparations with/without laser irradiation toward 4T1 cells (DOX, 7.5 μg/ml; ADA, 0.6 U/ml) (*n* = 3). (D) confocal laser scanning microscope (CLSM) images of 4T1 cells after incubation with PMAD or PPMAD for different times. Scale bars, 100 μm. (E) The semiquantitative analysis of DOX fluorescence intensity in (D). (F) Inverted microscope images of live/dead staining of 4T1 cells after treatment with PBS, free DOX, PPMA, or PPMAD in the absence or presence of irradiation of the NIR laser. Scale bars, 100 μm. *P < 0.05, **P < 0.01, and ***P < 0.001; N.S., *P* > 0.05 was tested via a Student’s *t* test.

First, the CCK8 method was used to investigate the biosafety of PPM. Figure 4A showed that the biocompatibility of PPM concentration in the range of 0 to 150 μg/ml was good, with a cell viability rate higher than 90% and no obvious effect on cell proliferation.

Afterward, the cellular uptake of PPMAD was assessed at 2, 4, and 6 h. As time elapsed, the fluorescence of PPMAD within the cells progressively intensified, suggesting a time-dependent pattern of cellular uptake. Moreover, the uptake capacity of tumor cells for MPDA modified with pHLIP peptide was markedly superior to that of the unmodified group, thereby substantiating the tumor-targeting potential of pHLIP peptide (Fig. 4D and E).

Subsequently, the impact of PPM on cell viability under NIR irradiation was investigated. The findings presented in Fig. 4B demonstrate that, when subjected to a power of 1.5 W/cm^2^, PPM impeded cell proliferation through the release of thermal energy. Notably, at a concentration of 75 μg/ml, evident cytotoxicity was observed, and at 100 μg/ml, the cell survival rate plummeted below 20%. Therefore, it can be inferred that a concentration of 75 μg/ml represents the critical threshold for effective eradication of tumor cells. Moreover, the escalation of PPM concentration significantly augmented the degree of inhibition exerted on tumor cells. These results corroborate previous research, suggesting that PPM solely exhibits tumor-killing effects at a specific concentration when coupled with NIR light, while simultaneously maintaining favorable biocompatibility in the absence of light irradiation.

To explore the impact of PPMAD on tumor cells, the efficacy of different formulation groups in inducing cell death was investigated. As depicted in Fig. 4C, while the free DOX group exhibited some antitumor effects compared to the complete blank group, the tumor inhibition rate did not show significant improvement under NIR light. However, the PPMAD group displayed a significantly enhanced tumor suppression rate under NIR light intervention, underscoring the indispensability of combined photothermal therapy and chemotherapy.

To validate the efficacy of photothermal therapy, a live-dead cell staining assay was conducted. Regardless of NIR light intervention, the green fluorescence remained predominant in the PBS group, whereas the fluorescence intensity in the free DOX group did not exhibit significant changes. In contrast, the red fluorescence in the PPMAD group exhibited a substantial enhancement, indicating that PPMAD amplifies the cytotoxic effect on tumor cells under NIR light (Fig. 4F).

### In vitro immunotherapy

**Fig. 5.**
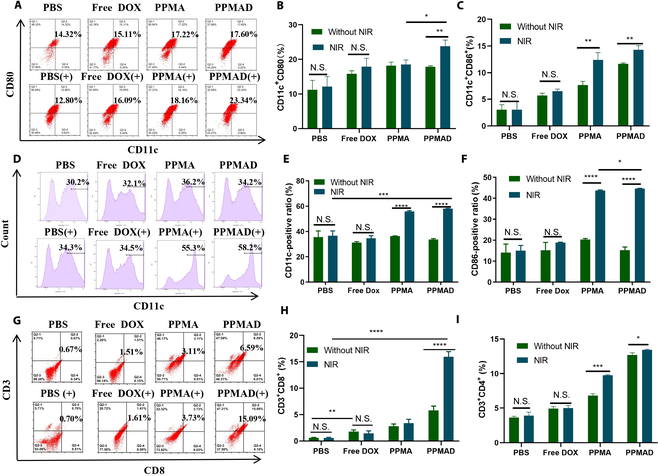
Analysis of DCs after coincubating with pretreated tumor cells. (A) Flow cytometry analysis of the expression of CD11c^+^CD80^+^CD86 on DCs after coincubation with pretreated tumor cells. (B and C) Ratios of CD11c^+^CD80^+^ and CD11^+^CD86^+^ DCs after coincubation with pretreated tumor cells. (D) Flow cytometry analysis of the expression of CD11c^+^ and (E and F) the percentages of CD11c^+^ and CD86^+^ DCs in the coincubation system and in the coincubation system containing NECA. (G) Flow cytometry analysis of expression of CD3^+^CD8^+^ and (H and I) ratios of CD3^+^CD8^+^ and CD3^+^CD4^+^ T cells in the ternary coincubation system. (+) represents plus laser treatment (808-nm NIR light irradiated at 1.5 W/cm^2^ for 8 min). *P < 0.05, **P <0.01, ***P < 0.001, and ****P < 0.0001; no significant difference (N.S.), *P* > 0.05 was tested via a Student's *t* test.

Next, the influence of PPMAD on the tumor cell microenvironment was examined, with specific attention given to the adenosinergic axis, a pivotal regulator of the tumor microenvironment. An important aspect of this investigation focused on the modulation of the immune capacity within the tumor microenvironment by PPMAD. DCs, crucial immune cells involved in the activation and differentiation of upstream lymphoid T cells, were utilized to evaluate the impact of PPMAD. BMDCs from mice were isolated and cocultured with colony-stimulating factors to induce their differentiation into DCs. Surface markers including CD11c, CD80, and CD86 were selected to characterize the maturation of DCs. Tumor cells undergoing ICD effects, such as up-regulation of CRT and release of high-mobility group box 1 and ATP, act as auxiliary stimuli for DC maturation. Therefore, 4T1 cells were exposed to different drugs (with or without laser treatment) and incubated for 24 h. Following this, immature DCs were added and coincubated for an additional 24 h, after which the biomarkers of mature DCs (CD11c, CD80, and CD86) were analyzed using flow cytometry. Figure 5A illustrates that compared to the PBS group, the CD11c^+^CD80^+^ ratio in the PPMAD(+) group exhibited a significant increase from 14.32% to 23.34%, which was notably higher than other groups (Fig. 5B and C).

Importantly, ADO existing within the tumor microenvironment can bind to the A2AR on the surface of DCs, leading to the inhibition of antigen presentation and DC maturation. The immunosuppressive effects of DCs can be alleviated by ADA, which converts the abundant ADO present at the tumor site into inosine. To validate the impact of ADA on the immune response, a certain amount of 5′-*N*-ethylcarboxamidoadenosine (NECA) (an ADO mimic) was introduced into the coincubation system to mimic the conditions within the tumor microenvironment and evaluate DC maturation. As illustrated in Fig. 5D, the free DOX group only exhibited a slight increase in the positive rate of CD11c, whereas the expression levels of CD11c and CD80 in the PPMAD group and laser group were significantly higher compared to the other groups (Fig. 5E and F). The CD11c ratio in the PPMAD(+) group increased by 1.92 times in comparison to the PBS group. These findings indicate that the presence of NECA impedes DC maturation, but the action of ADA effectively mitigates this phenomenon.

In the presence of ICD effects induced by tumor cells, mature DCs are capable of presenting antigens to naive T cells, leading to their differentiation into cytotoxic T cells or regulatory T cells (T_reg_ cells), thus initiating an immune response. To investigate this phenomenon, a 3-way coincubation system consisting of 4T1 cells (pretreated with different drugs or without heat), immature DCs, and lymphocytes was established. Following 48 h of culture, the proliferation of CD3^+^CD4^+^ and CD3^+^CD8^+^ T cells was analyzed. As depicted in Fig. 5G, when 4T1 cells were coincubated with PPMAD and subjected to laser-triggered hyperthermia, the expression levels of CD3^+^CD4^+^ and CD3^+^CD8^+^ were significantly augmented compared to the other groups. The free DOX group exhibited some tumor-killing effects and triggered an ICD response, albeit with a lower magnitude compared to the PPMAD(+) due to the absence of targeting and a photothermal agent. The disparity between the free DOX group and the group without light intervention was negligible (Fig. 5H and I). The CD3^+^CD8^+^ level in the PPMAD(+) group reached as high as 15.09%, whereas it remained below 1% in the PBS group. These findings indicate that PPMAD, under the influence of laser intervention, can induce a substantial ICD response, initiating a cascade of reactions that affect DC maturation. Ultimately, this promotes the differentiation of naive T cells into cytotoxic T cells, diminishes the formation of T_reg_ cells, and reverses the immunosuppressive state within the tumor microenvironment. The ADO content in the supernatant from the cell coincubation was assessed using an ELISA kit. The results showed that the ADO level in the PPMAD(+) group was lower than that in the other groups, indicating that ADA effectively degraded ADO, thereby improving the tumor microenvironment (Fig. S3).

### In vivo distribution

Indocyanine green was used as a fluorescence probe to investigate the in vivo distribution of PPM. Free indocyanine green was rapidly eliminated and showed systemic distribution without effective tumor targeting. Within 24 h, it was completely metabolized with no remaining fluorescence. In contrast, indocyanine green loaded into MPDA exhibited excellent tumor targeting ability and prolonged retention due to the enhanced permeability and retention effect. They showed maximum fluorescence at 24 h, with a significant portion distributed within tumor tissues. Even after 48 h, strong fluorescence was still observed. Moreover, pHLIP-modified MPDA demonstrated higher fluorescence distribution at the tumor site compared to nanoparticles without pHLIP modification, due to the tumor tropism of pHLIP (Fig. S4).

After 48 h of intravenous injection, the mice were dissected, and their organs were isolated to assess the fluorescence intensity between them. As illustrated in Figs. S5 and S6, the fluorescence of free indocyanine green considerably diminished after 48 h of metabolic processes within the body, with the majority of it being distributed in the liver. In contrast, MPDA loaded with indocyanine green retained a significant level of fluorescence even after 48 h, accumulating in substantial quantities at the tumor site. Remarkably, pHLIP-modified MPDA exhibited heightened fluorescence intensity.

### In vivo antitumor effect

**Fig. 6.**
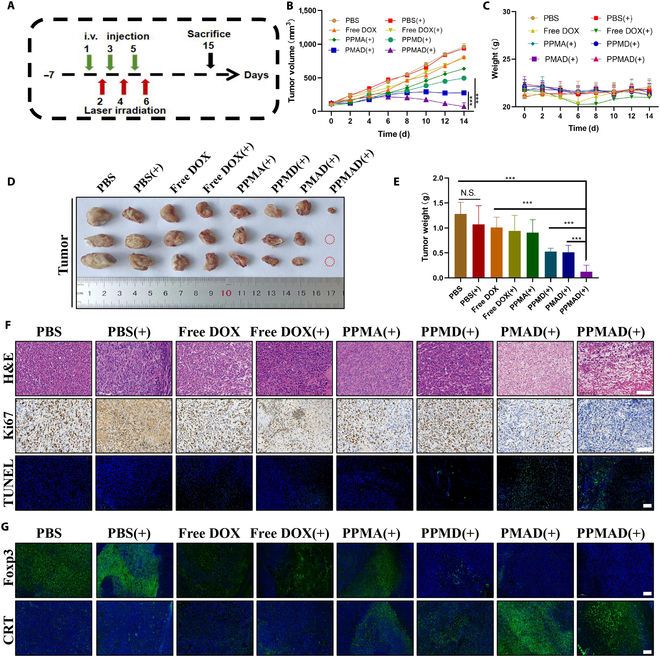
In vivo antitumor efficacy in 4T1-tumor-bearing models. (A) Schematic of the treatment regimen. i.v., intravenous. (B) Curves showing tumor volumes of mice after various treatments (DOX, 5 mg/kg; ADA, 400 U/kg). (C) Change curves of mouse weights after various treatments (*n* = 6). (D) Representative photographs of harvested tumors after different treatments. (E) Weights of tumor in different treatment groups. (F) The representative images of tumors in each group. Histopathology in tumors was identified using H&E staining and Ki67 staining. Histology in the tumor was identified using immunofluorescence staining for TUNEL. Scale bars, 100 μm. (G) Immunofluorescence was used to examine the levels of Foxp3^+^ and CRT^+^ in spleen sections at the end of the observation period. Scale bars, 200 μm. ***P < 0.001; no significant difference (N.S.), *P* > 0.05 was tested via a Student's *t* test.

Afterward, an in vivo evaluation of the antitumor effect of PPMAD was conducted (Fig. 6A). After 7 d, the tumor-bearing mice received intravenous administration every 2 d, with laser-induced hyperthermia administered on the day following the first dose. Figure 6B illustrated the changes in tumor volume among the mice. It was evident that the tumor volume in each group decreased to varying degrees compared to the PBS group, indicating a certain level of tumor suppression. Notably, there was no significant difference in tumor volume between the PBS group and the PBS(+) group, with the latter having a volume of approximately 1,000 mm^3^. This suggested that laser irradiation alone could not produce an effective tumor-killing effect. However, after PPMAD(+) treatment, the tumor volume decreased to less than 200 mm^3^, resulting in a tumor inhibition rate of more than 80% and demonstrating a strong antitumor effect.

The bodyweight of tumor-bearing mice throughout the treatment period was shown in Fig. 6C. No significant fluctuations were observed. Following the completion of the entire treatment cycle, the mice were dissected in vitro to examine the tumor weight. The results revealed that the tumors in each group had reduced to varying degrees, with the PPMAD group showing the most significant reduction. In fact, the tumors had almost completely disappeared in this group, and the tumor weight was the lowest among all the groups (Fig. 6D and E).

To gain further insights into the molecular mechanism underlying PPMAD’s antitumor resistance, immunofluorescence and immunohistochemical staining were performed on animal spleens and tumors. H&E staining and sectioning of the tumor revealed notable enlargement of the nucleoli, distorted nuclei, and tissue rupture in the PPMAD plus laser group, indicating a strong antitumor efficacy.

Immunohistochemistry and immunofluorescence assays targeting cell proliferation and apoptosis were subsequently conducted on tumor tissues. The expression of the proliferation marker Ki67 was higher in the control group and lower in the PPMAD plus laser group, indicating that PPMAD effectively inhibited tumor cell proliferation under laser intervention. The apoptosis experiment confirmed this, with significantly higher levels of apoptosis observed in the PPMAD plus laser group compared to the other groups (Fig. 6F).

The expression level of the T_reg_ cell marker FOXp3 was higher in the PBS group and the PBS plus laser group but lower in the PPMAD plus laser group, suggesting that the PPMAD plus laser group exhibited lower degrees of immunosuppression than the control group. In addition, the level of CRT in the PPMAD plus laser group was significantly higher than in the other groups, indicating that PPMAD effectively induced the ICD effect of tumor cells under laser intervention. This further regulated and improved the immune status of the tumor microenvironment, reversing the adverse situation of immunosuppression (Fig. 6G).

### In vivo immunotherapy

**Fig. 7.**
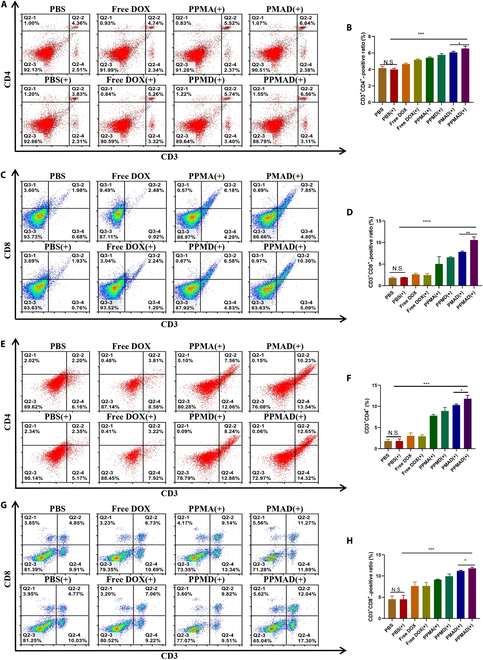
Assessment of immune response after different treatments in a 4T1 tumor model using flow cytometry. (A) The expression of a CD3^+^CD4^+^ and (B) CD3^+^CD8^+^ T cells in the spleen were analyzed by flow cytometry at the end of the observation period. (C) Percentages of CD3^+^CD4^+^ and (D) CD3^+^CD8^+^ T cells in spleen were calculated on the basis of (A) and (B). (E) The expression of a CD3^+^CD4^+^ and (F) CD3^+^CD8^+^ T cells in tumors were analyzed by flow cytometry at the end of the observation period. (G) Percentages of CD3^+^ CD4^+^ and (H) CD3^+^CD8^+^ T cells in tumor were calculated on the basis of (G) and (H). *P < 0.05, **P <0.01, ***P <0.001, and ****P < 0.0001; no significant difference (N.S.), *P* > 0.05 was tested via a Student's *t* test.

We further investigated the immunological mechanisms of PPMAD against tumors and its ability to induce immune responses in vivo. Flow cytometry analysis revealed a noteworthy increase in the expression levels of CD3^+^CD4^+^ and CD3^+^CD8^+^ in the PPMAD plus laser group in spleen cell (Fig. 7A and B). These findings hold significance as cytotoxic T cells play a pivotal role in inhibiting tumor growth, while T helper cells are crucial for establishing and regulating adaptive immunity, making them integral components of tumor immunotherapy (Fig. 7C and D).

Quantification of the primary immune complexes of T cells in the tumor of mice at the conclusion of the treatment cycle validated these results. As depicted in Fig. 7E and F, the PPMAD plus laser group exhibited a substantial elevation in CD3^+^CD4^+^ and CD3^+^CD8^+^ expression levels within the tumor, with the other groups also displaying varying degrees of improvement compared to the PBS group (Fig. S7). However, these improvements were comparatively less pronounced than those observed in the PPMAD plus laser group. These findings indicate that the PPMAD plus laser group effectively enhanced the immunosuppressive status of the tumor microenvironment (Fig. 7G and H). Following the grinding and homogenization of tumor tissues, we observed that the ADO level in the PPMAD(+) group was notably lower than in the other groups. Specifically, it was approximately 50% of the ADO content found in the PBS group. This indicates that PPMAD effectively enhanced the local immune microenvironment of tumors by substantially reducing ADO levels (Fig. S8).

**Fig. 8.**
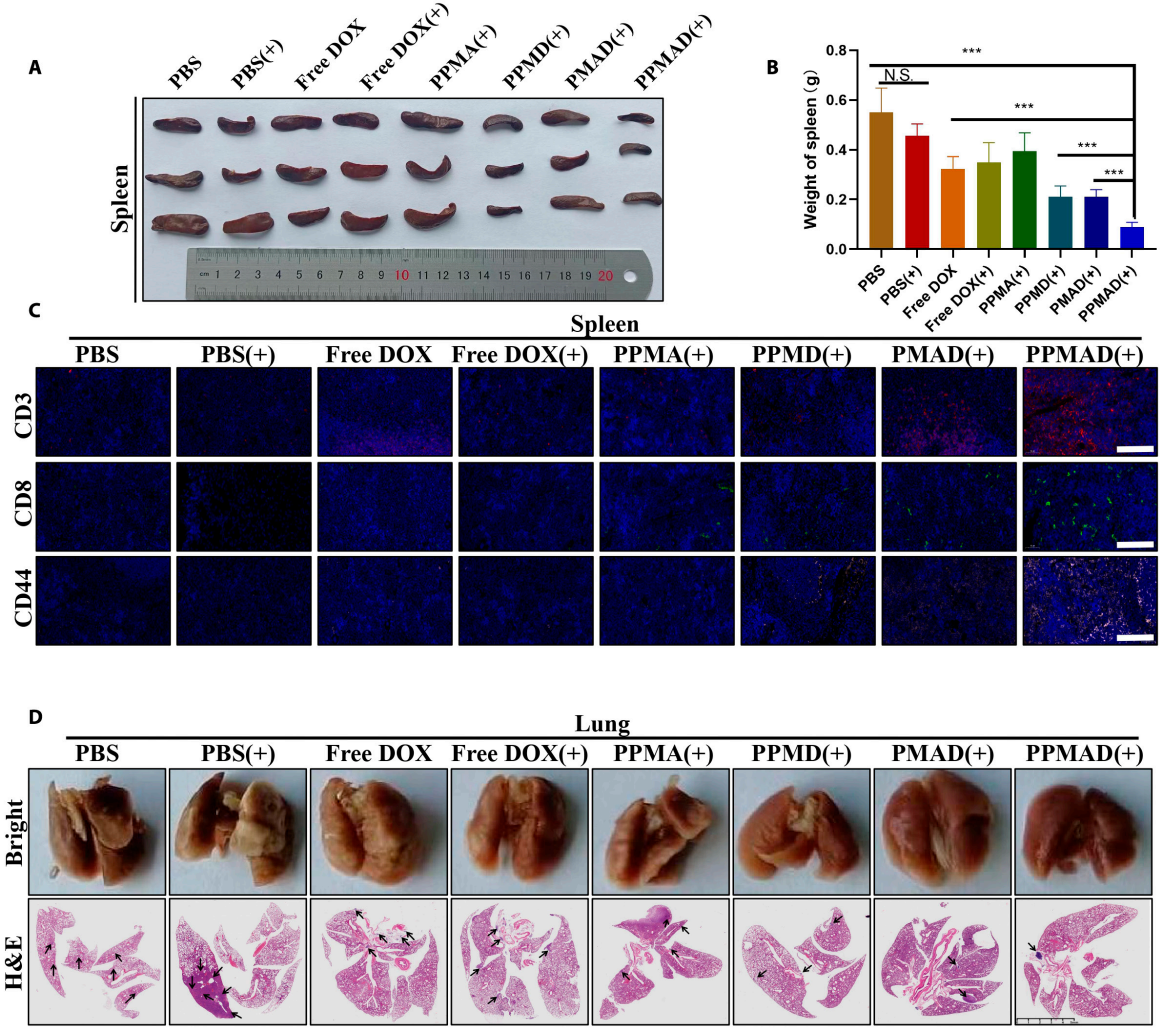
Evaluation of the immune response after different treatments in 4T1 tumor models. (A) The representative images of spleen in each group. (B) Weights of the spleen in different treatment groups (*n* = 6). (C) Histology in the spleen was identified using immunofluorescence staining for CD3^+^, CD8^+^, and CD44^+^. Scale bars, 100 μm. (D) Histopathology in the lung was identified using H&E staining. Scale bar, 5 mm. ***P < 0.001; no significant difference (N.S.), *P* > 0.05 was tested via a Student's *t* test.

The spleen plays a crucial role in immune activation. When immune activation is incomplete, the spleen tends to swell, indicating immune activation disorder. The weight of the spleen in each group was measured to assess the immune activation status. The results showed that the immune activation status was significantly improved in all groups after treatment and the immunosuppression status was also enhanced compared to the PBS group. The PPMAD group exhibited the lowest spleen swelling and the lightest spleen weight, indicating a significantly better immune status than the other groups (Fig. 8A and B). The spleen was subjected to immunofluorescence staining after sectioning. The results showed significantly higher expression levels of CD3^+^CD8^+^ in the PPMAD plus laser group compared to the other groups (Fig. 8C), indicating a significantly improved immune environment. Further investigation of CD44, closely associated with tumor invasion and metastasis, revealed higher levels in the PBS group but limited expression in the PPMAD plus laser group. These findings demonstrated that the PPMAD plus laser group exhibited strong antitumor and antimetastasis effects, effectively enhancing the body’s immune ability, improving the weakened immune system caused by the tumor itself, and exerting an antitumor effect [30].

In comparison to the PBS group, the PPMAD group in combination with hyperthermia exhibited a more robust capacity to inhibit tumor metastasis. Conversely, the free DOX group displayed only modest reduction in tumor metastasis and recurrence. In summary, PPMAD with laser hyperthermia demonstrated remarkable antitumor efficacy and effectively prevented tumor metastasis to the lungs (Fig. 8D).

The in vivo results demonstrate that PMAD effectively delivers drugs to the tumor site. With laser intervention, DOX release is enhanced, leading to chemotherapy. In addition, the laser induces a strong photothermal synergistic effect that results in tumor ablation and activates ICD yielding a significant therapeutic impact. However, the issue of immunosuppression within the tumor microenvironment remained unresolved until the timely intervention of ADA. Flow cytometry and immunofluorescence experiments indicated that the immune microenvironment shifted from immunosuppressive to positive, facilitating more active tumor treatment. Notably, CD3^+^ and CD8^+^ cytotoxic T cells were predominant, while Foxp3^+^ immunosuppressive T_reg_ cells were significantly diminished, which is consistent with in vitro experiments.

### Biosafety

Last, the safety of PPMAD on the body was assessed. Sections of animal organs were stained, and the tissue sections revealed no significant pathological changes in the organs (Fig. S9). Comparisons of biochemical indexes and routine blood tests in animal blood with the control group showed no evident abnormalities in the blood biochemical indexes [ALT and creatinine (CR)], and the main blood parameters remained within a reasonable range (Figs. S10 and S11). These findings indicated that PPMAD demonstrated excellent biosafety in vivo and held great potential for broad applications.

## Conclusion

In summary, we have developed modified nanoparticles called PPMADs to transport DOX and ADO scavengers. PPMAD demonstrates excellent selectivity for tumor sites due to the presence of pHLIP, resulting in a high concentration of the drug at the tumor site. When laser irradiation is applied, heat is generated at the tumor location, which enhances drug release and leads to tumor eradication. DOX triggers ICD, which promotes the maturation, differentiation, and antigen presentation of DCs. In addition, ADO scavengers convert ADO into inosine, reducing immunosuppression and stimulating T cell activity. Overall, our specially designed nanoadjuvants effectively overcome the challenge of ADO accumulation caused by ICD, while also providing sufficient energy to T cells. This approach holds great promise for a combined strategy involving phototherapy, chemotherapy, and immunotherapy.

## Data Availability

All data of this study are available from the corresponding authors upon reasonable request.

## References

[1] Derakhshan F, Reis-Filho JS. Pathogenesis of triple-negative breast cancer. Annu Rev Pathol. 2022;17:181–204. 35073169 10.1146/annurev-pathol-042420-093238 PMC9231507

[2] Yin L, Duan JJ, Bian XW, Yu SC. Triple-negative breast cancer molecular subtyping and treatment progress. Breast Cancer Res. 2020;22(1):61. 32517735 10.1186/s13058-020-01296-5 PMC7285581

[3] Li Y, Zhang H, Merkher Y, Chen L, Liu N, Leonov S, Chen Y. Recent advances in therapeutic strategies for triple-negative breast cancer. J Hematol Oncol. 2022;15(1):121. 36038913 10.1186/s13045-022-01341-0 PMC9422136

[4] Yang F, Xiao Y, Ding JH, Jin X, Ma D, Li DQ, Shi JX, Huang W, Wang YP, Jiang YZ, et al. Ferroptosis heterogeneity in triple-negative breast cancer reveals an innovative immunotherapy combination strategy. Cell Metab. 2023;35(1):84–100.e8. 36257316 10.1016/j.cmet.2022.09.021

[5] Riley RS, June CH, Langer R, Mitchell MJ. Delivery technologies for cancer immunotherapy. Nat Rev Drug Discov. 2019;18(3):175–196. 30622344 10.1038/s41573-018-0006-z PMC6410566

[6] Zhang Y, Zhang Z. The history and advances in cancer immunotherapy: Understanding the characteristics of tumor-infiltrating immune cells and their therapeutic implications. Cell Mol Immunol. 2020;17(8):807–821. 32612154 10.1038/s41423-020-0488-6 PMC7395159

[7] Baxevanis CN, Perez SA, Papamichail M. Cancer immunotherapy. Crit Rev Clin Lab Sci. 2009;46(4):167–189. 19650714 10.1080/10408360902937809

[8] Morrison AH, Byrne KT, Vonderheide RH. Immunotherapy and prevention of pancreatic cancer. Trends Cancer. 2018;4(6):418–428. 29860986 10.1016/j.trecan.2018.04.001 PMC6028935

[9] Ahmed A, Tait SWG. Targeting immunogenic cell death in cancer. Mol Oncol. 2020;14(12):2994–3006. 33179413 10.1002/1878-0261.12851 PMC7718954

[10] Duan X, Chan C, Lin W. Nanoparticle-mediated immunogenic cell death enables and potentiates cancer immunotherapy. Angew Chem Int Ed Engl. 2019;58(3):670–680. 30016571 10.1002/anie.201804882 PMC7837455

[11] Moriyama K, Nishida O. Targeting cytokines, pathogen-associated molecular patterns, and damage-associated molecular patterns in sepsis via blood purification. Int J Mol Sci. 2021;22(16):8882. 34445610 10.3390/ijms22168882 PMC8396222

[12] Zhou M, Aziz M, Wang P. Damage-associated molecular patterns as double-edged swords in sepsis. Antioxid Redox Signal. 2021;35(15):1308–1323. 33587003 10.1089/ars.2021.0008 PMC8817718

[13] Franco R, Lillo A, Navarro G, Reyes-Resina I. The adenosine A2A receptor is a therapeutic target in neurological, heart and oncogenic diseases. Expert Opin Ther Targets. 2022;26(9):791–800. 36300278 10.1080/14728222.2022.2136570

[14] Bao X, Xie L. Targeting purinergic pathway to enhance radiotherapy-induced immunogenic cancer cell death. J Exp Clin Cancer Res. 2022;41(1):222. 35836249 10.1186/s13046-022-02430-1 PMC9284706

[15] Yegutkin GG, Boison D. ATP and adenosine metabolism in cancer: Exploitation for therapeutic gain. Pharmacol Rev. 2022;74(3):797–822. 35738682 10.1124/pharmrev.121.000528 PMC9553103

[16] Vaupel P, Multhoff G. Revisiting the Warburg effect: Historical dogma versus current understanding. J Physiol. 2021;599(6):1745–1757. 33347611 10.1113/JP278810

[17] Payen VL, Mina E, Van Hée VF, Porporato PE, Sonveaux P. Monocarboxylate transporters in cancer. Mol Metab. 2020;33:48–66. 31395464 10.1016/j.molmet.2019.07.006 PMC7056923

[18] Wang ZH, Peng WB, Zhang P, Yang XP, Zhou Q. Lactate in the tumour microenvironment: From immune modulation to therapy. EBioMedicine. 2021;73: Article 103627. 34656878 10.1016/j.ebiom.2021.103627 PMC8524104

[19] Jadidi-Niaragh F, Atyabi F, Rastegari A, Kheshtchin N, Arab S, Hassannia H, Ajami M, Mirsanei Z, Habibi S, Masoumi F, et al. CD73 specific siRNA loaded chitosan lactate nanoparticles potentiate the antitumor effect of a dendritic cell vaccine in 4T1 breast cancer bearing mice. J Control Release. 2017;246:46–59. 27993599 10.1016/j.jconrel.2016.12.012

[20] Qi J, Jin F, You Y, du Y, Liu D, Xu X, Wang J, Zhu L, Chen M, Shu G, et al. Synergistic effect of tumor chemo-immunotherapy induced by leukocyte-hitchhiking thermal-sensitive micelles. Nat Commun. 2021;12(1):4755. 34362890 10.1038/s41467-021-24902-2 PMC8346467

[21] Zhulai G, Oleinik E, Shibaev M, Ignatev K. Adenosine-metabolizing enzymes, adenosine kinase and adenosine deaminase, in cancer. Biomol Ther. 2022;12(3):418. 10.3390/biom12030418 PMC8946555 35327609

[22] Wang T, Gnanaprakasam JNR, Chen X, Kang S, Xu X, Sun H, Liu L, Rodgers H, Miller E, Cassel TA, et al. Inosine is an alternative carbon source for CD8^+^-T-cell function under glucose restriction. Nat Metab. 2020;2(7):635–647. 32694789 10.1038/s42255-020-0219-4 PMC7371628

[23] Samami E, Aleebrahim-Dehkordi E, Mohebalizadeh M, Yaribash S, Saghazadeh A, Rezaei N. Inosine, gut microbiota, and cancer immunometabolism. Am J Physiol Endocrinol Metab. 2023;324(1):E1–E8. 36416582 10.1152/ajpendo.00207.2022

[24] Liu Y, Ai K, Liu J, Deng M, He Y, Lu L. Dopamine-melanin colloidal nanospheres: An efficient near-infrared photothermal therapeutic agent for in vivo cancer therapy. Adv Mater. 2013;25(9):1353–1359. 23280690 10.1002/adma.201204683

[25] Tian Q, Jiang F, Zou R, Liu Q, Chen Z, Zhu M, Yang S, Wang J, Wang J, Hu J. Hydrophilic Cu9S5 nanocrystals: A photothermal agent with a 25.7% heat conversion efficiency for photothermal ablation of cancer cells in vivo. ACS Nano. 2011;5(12):9761–9771. 22059851 10.1021/nn203293t

[26] Zhang L, Yang P, Guo R, Sun J, Xie R, Yang W. Multifunctional mesoporous polydopamine with hydrophobic paclitaxel for photoacoustic imaging-guided chemo-photothermal synergistic therapy. Int J Nanomedicine. 2019;14:8647–8663. 31806962 10.2147/IJN.S218632 PMC6839591

[27] Wang L, He Y, He T, Liu G, Lin C, Li K, Lu L, Cai K. Lymph node-targeted immune-activation mediated by imiquimod-loaded mesoporous polydopamine based-nanocarriers. Biomaterials. 2020;255: Article 120208. 32569862 10.1016/j.biomaterials.2020.120208

[28] Chen F, Xing Y, Wang Z, Zheng X, Zhang J, Cai K. Nanoscale polydopamine (PDA) meets π-π interactions: An interface-directed coassembly approach for mesoporous nanoparticles. Langmuir. 2016;32(46):12119–12128. 27933877 10.1021/acs.langmuir.6b03294

[29] Zhang Y, Dang M, Tian Y, Zhu Y, Liu W, Tian W, Su Y, Ni Q, Xu C, Lu N, et al. Tumor acidic microenvironment targeted drug delivery based on pHLIPs-modified mesoporous organosilica nanoparticles. ACS Appl Mater Interfaces. 2017;9(36):30543–30552. 28809111 10.1021/acsami.7b10840

[30] Hassn Mesrati M, Syafruddin SE, Mohtar MA, Syahir A. CD44: A multifunctional mediator of cancer progression. Biomol Ther. 2021;11(12):1850. 10.3390/biom11121850 PMC8699317 34944493

