# Supplementary material for: Polydopamine-based Nanoadjuvants Promote a Positive Feedback Loop for Cancer Immunotherapy via Overcoming Photothermally Boosted T Cell Exhaustion
Source: Biomater Res. 2025 Mar 19;29:0166. doi: 10.34133/bmr.0166 (PMC11922554; doi:10.34133/bmr.0166)
Supplement: Supplementary 1 — Figs. S1 to S11 [file bmr.0166.f1.docx]

Supplementary Information

Xiao-Kai Chi**^1,2,3^**, Hai-Rui Zhang**^1^**, Jing-Jing Gao**^1^**, Jin Su**^2*^**, Yong-Zhong Du^3^**^*^,** Xiao-Ling Xu^1^**^*^**

^1^Shulan International Medical College, Zhejiang Shuren University, Hangzhou, 310015, PR China.

^2^[College of Pharmacy, Jiamusi University, Jiamusi, 154007, PR China](https://scholar.cnki.net/home/search?sw=7&sw-input=College%20of%20Pharmacy,%20Jiamusi%20University,%20Jiamusi%20154007,%20China).

^3^Institute of Pharmaceutics, College of Pharmaceutical Sciences, Zhejiang University, Hangzhou 310058, PR China;

Correspondence to:

Dr. X. L. Xu (Shulan International Medical College, Zhejiang Shuren University), 8 Shuren Street, Hangzhou 310015, China; Email:[ziyao1988@zju.edu.cn](mailto:ziyao1988@zju.edu.cn)

Dr. Y. Z. Du (Institute of Pharmaceutics, College of Pharmaceutical Sciences, Zhejiang University), 866 Yu-Hang-Tang Road, Hangzhou, 310058, China. Tel: +86-571-88208435; Fax: +86-571-88208435. E-mail: [duyongzhong@zju.edu.cn;](mailto:duyongzhong@zju.edu.cn;)

Dr. J. Su (College of Pharmacy, Jiamusi University), 258 Xuefu Road, Jiamusi 154007, China. Email: sujin@jmsu.edu.cn


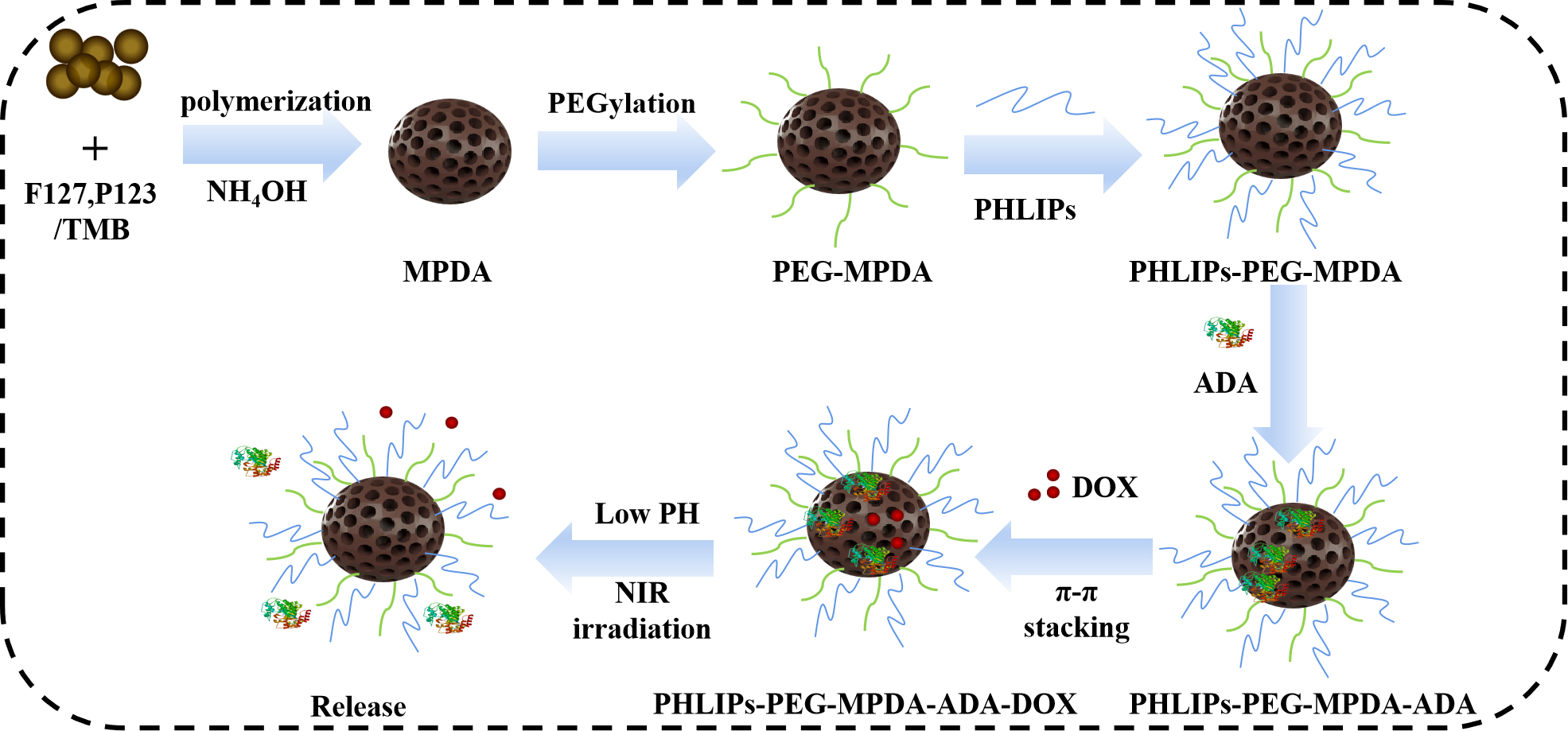


**Fig. S1.**Preparation process of PPMAD and its release process.


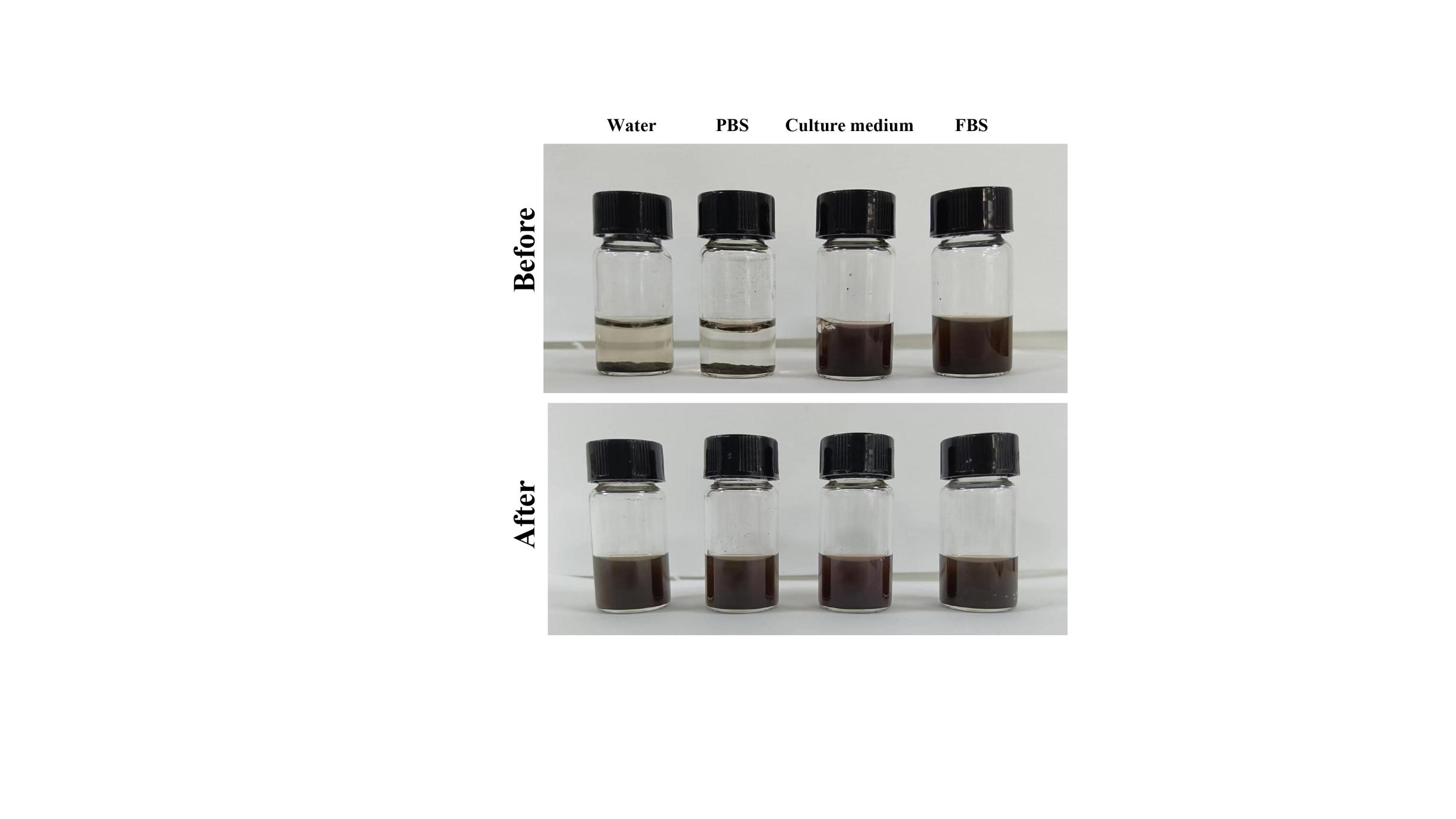


**Fig. S2** Photographs of MPDA before and after PEG modified in different media (Water, PBS, RPMI-1640 medium (containing 10% FBS), FBS) for 8hrs.


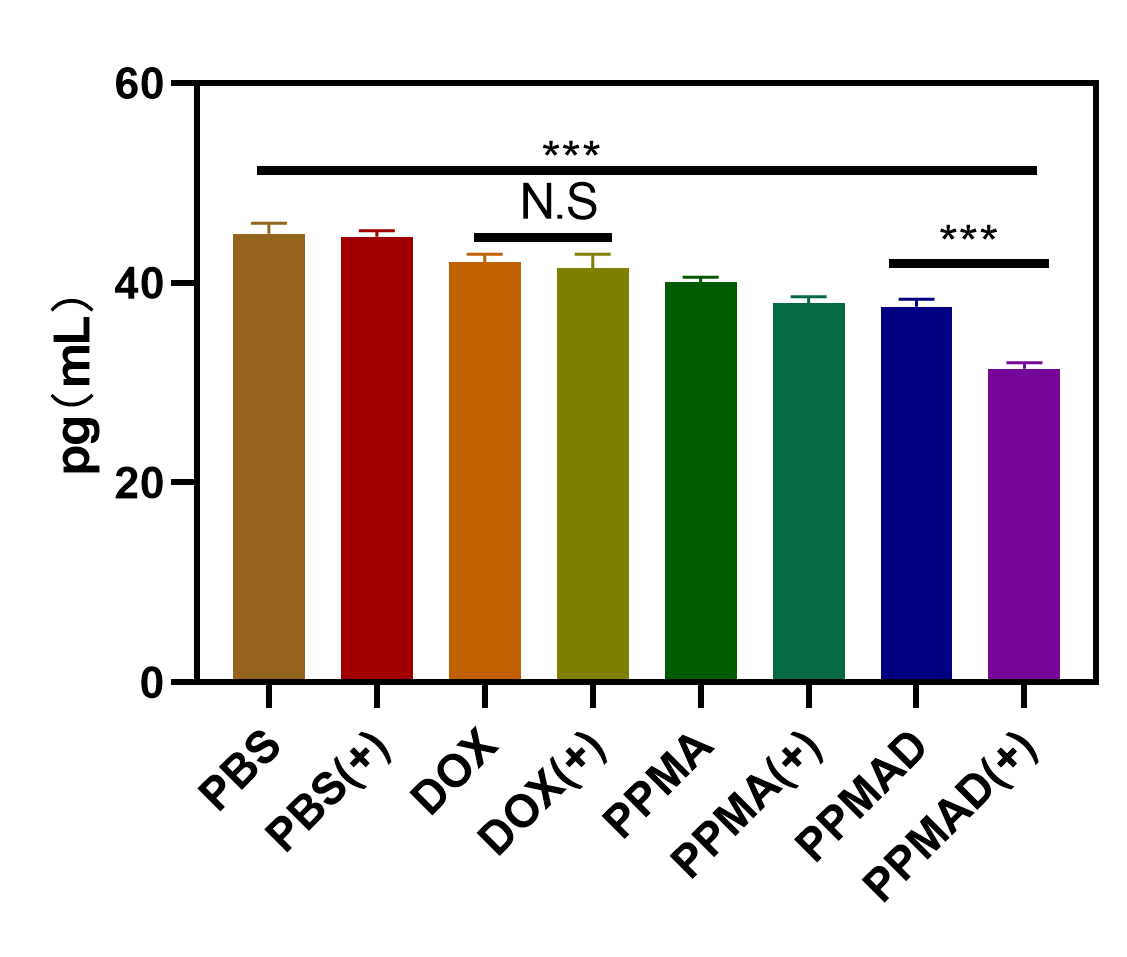


**Fig. S3** Adenosine content in the cell co-incubation solution.


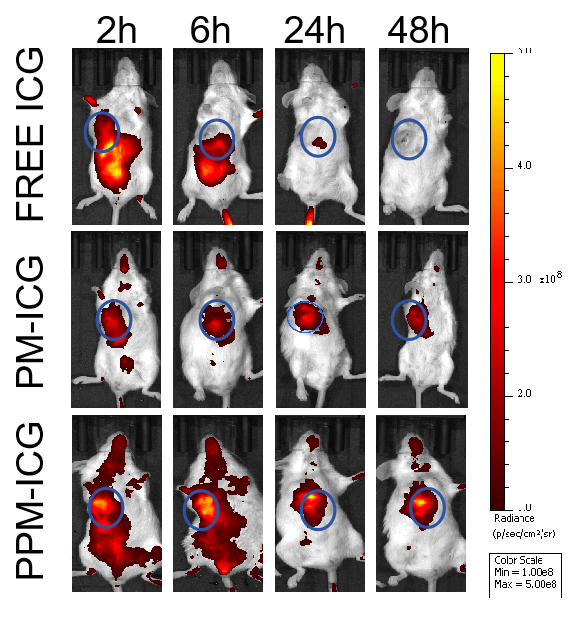


**Fig. S4** Imaging of tumor-bearing nude mice treated with Free ICG, PPM-ICG,PM-ICG at different pointin-times.


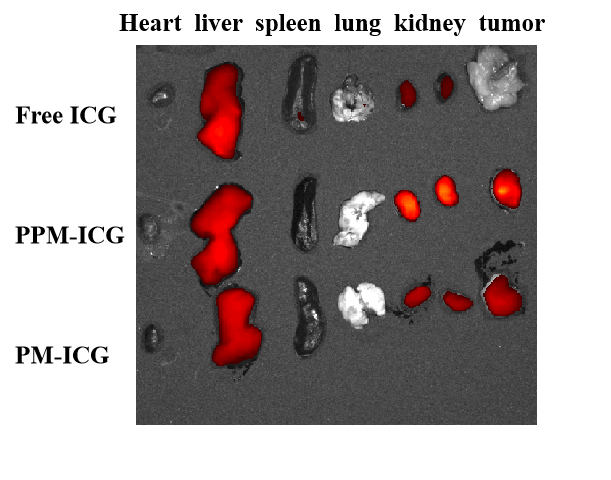


**Fig. S5** Imaging of major organs in tumor-bearing mice injected intravenously with Free ICG,PPM-ICG and PM-ICG at 48h.


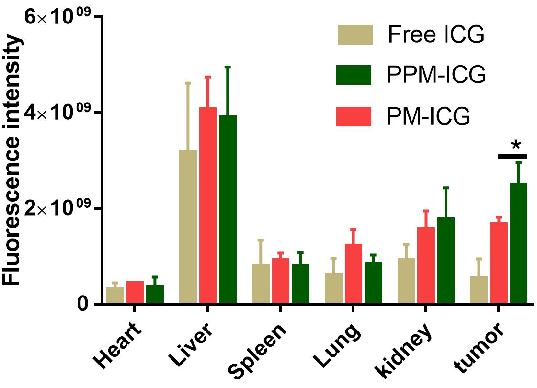


**Fig. S6** The semi-quantitative analysis of fluorescence intensity in tumor and tissues of Fig. S5.


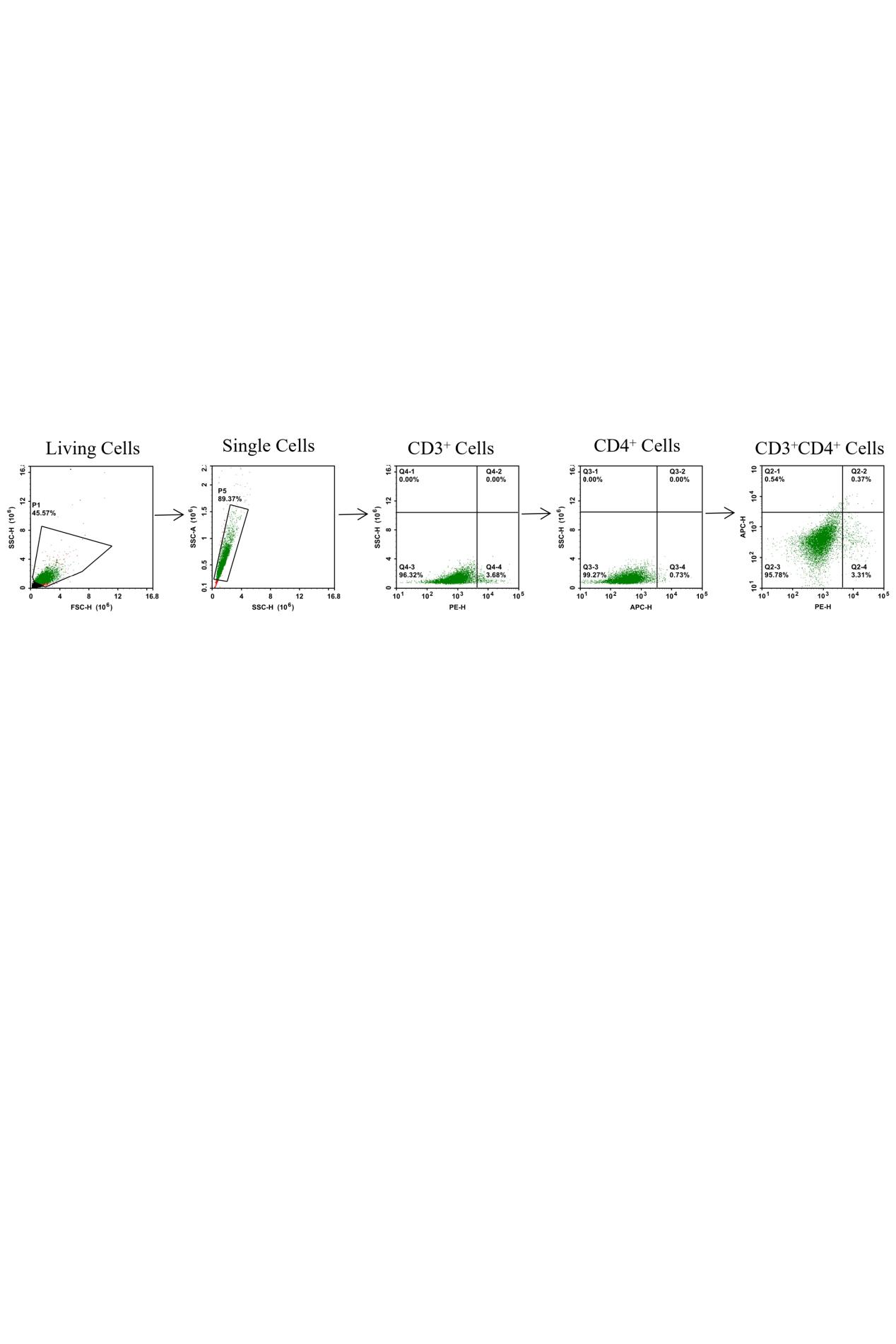


**Fig. S7** Lymphotic T cell gating strategy for flow cytometry.


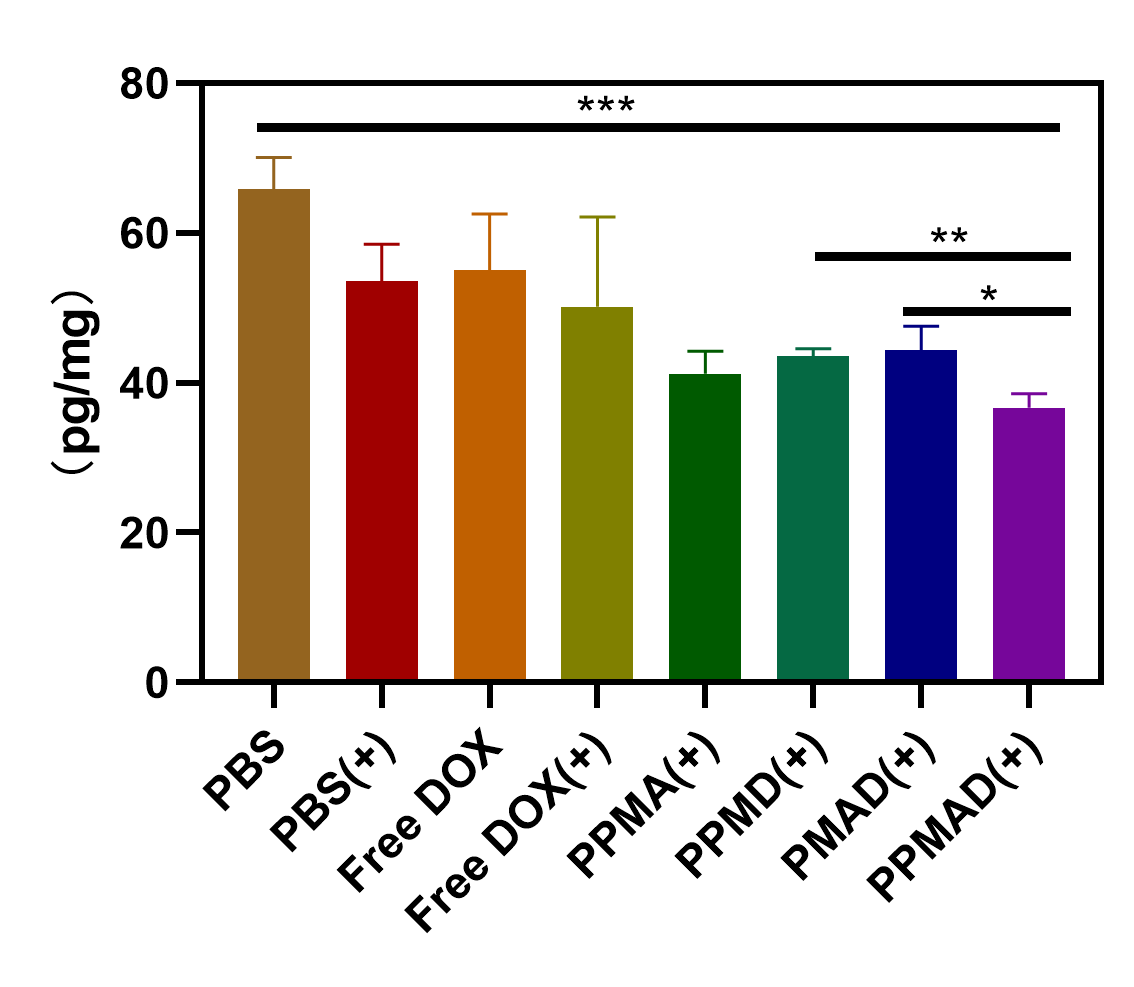


**Fig.S8** Adenosine content in the tumor


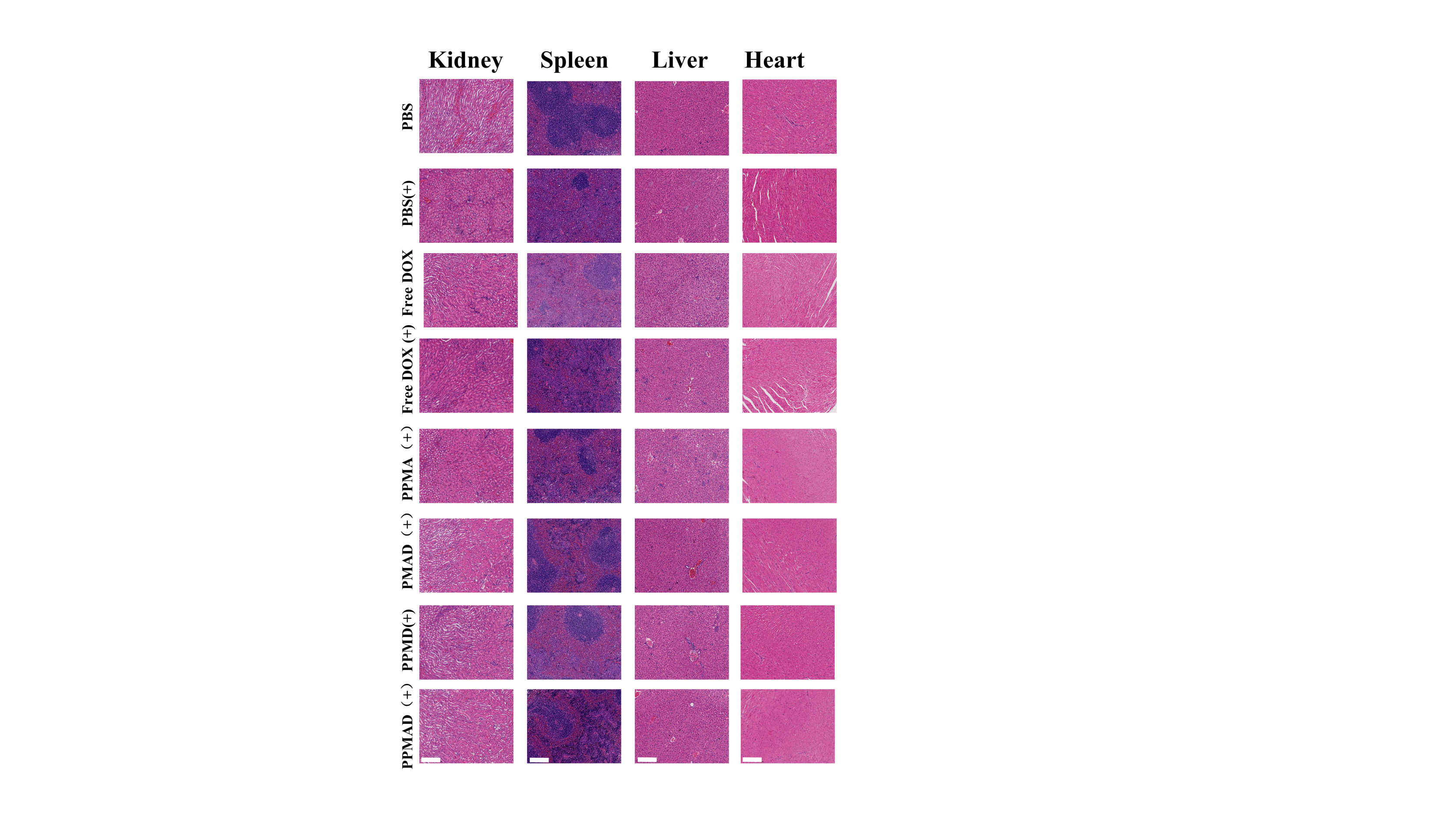


**Fig. S9** H&E staining of the sections of main organs including liver, kidney, spleen, and heart from the mice after different treatment.Scale bar:200um.


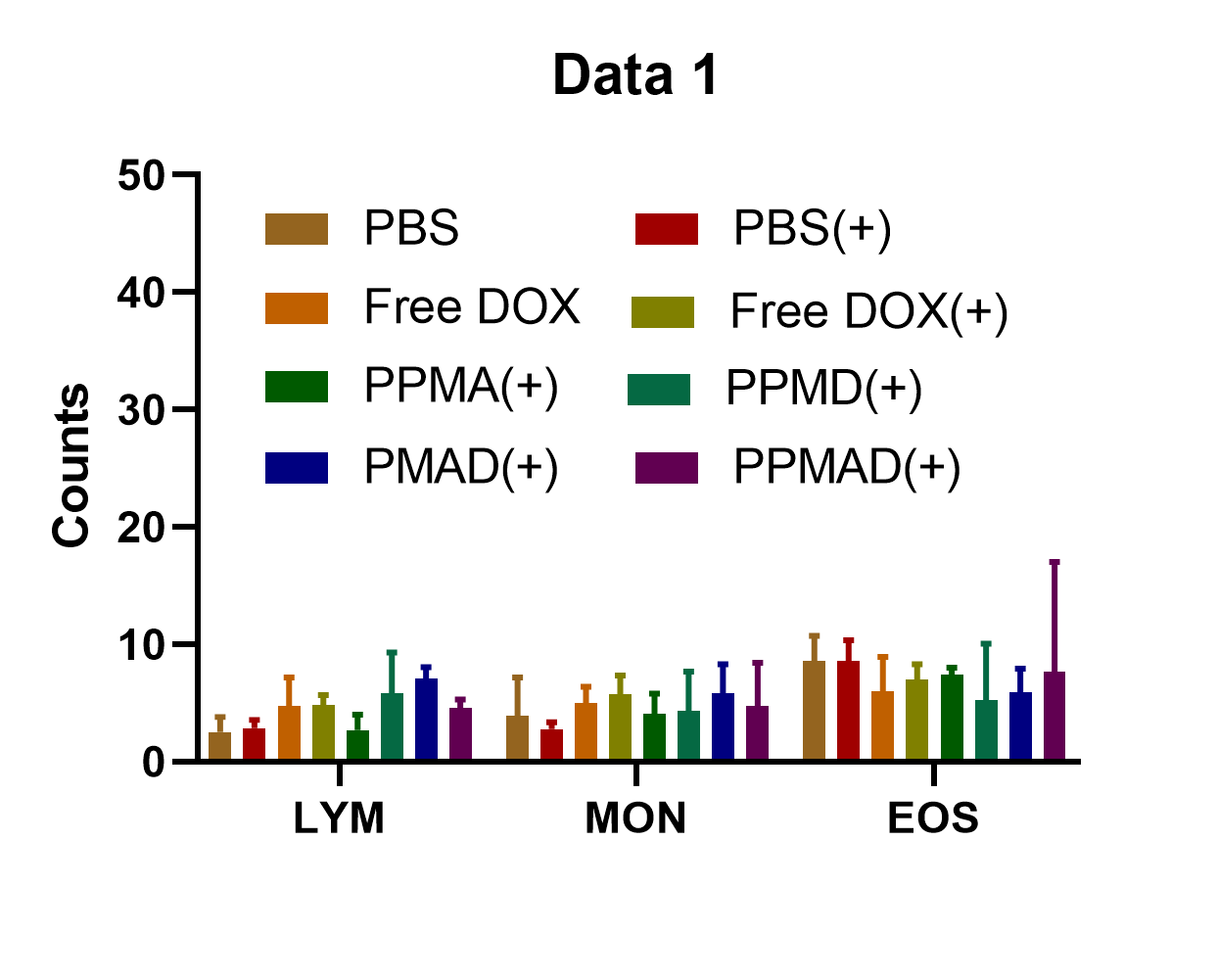

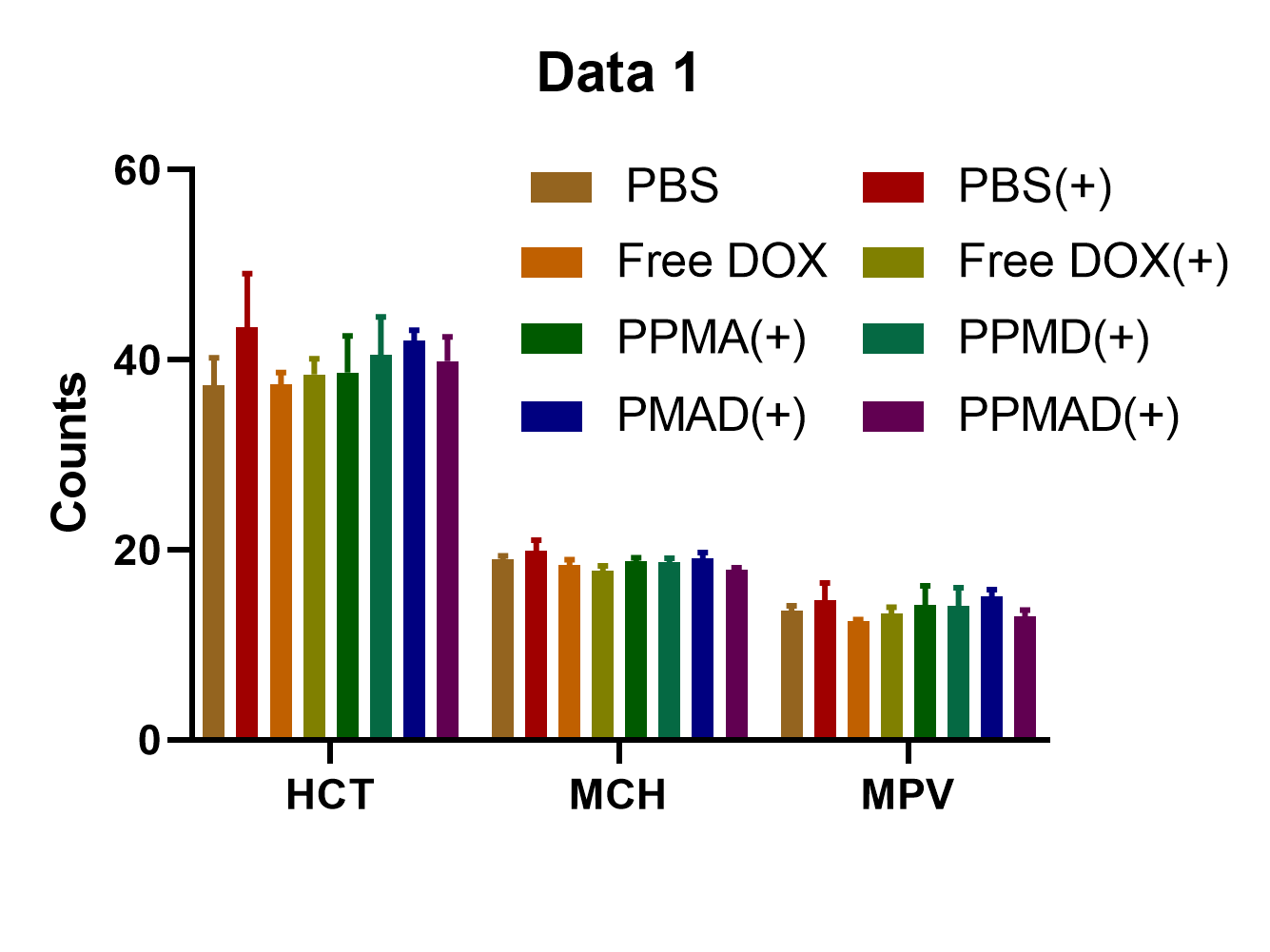


**Fig. S10** blood routine analysis of mice after different treatment .


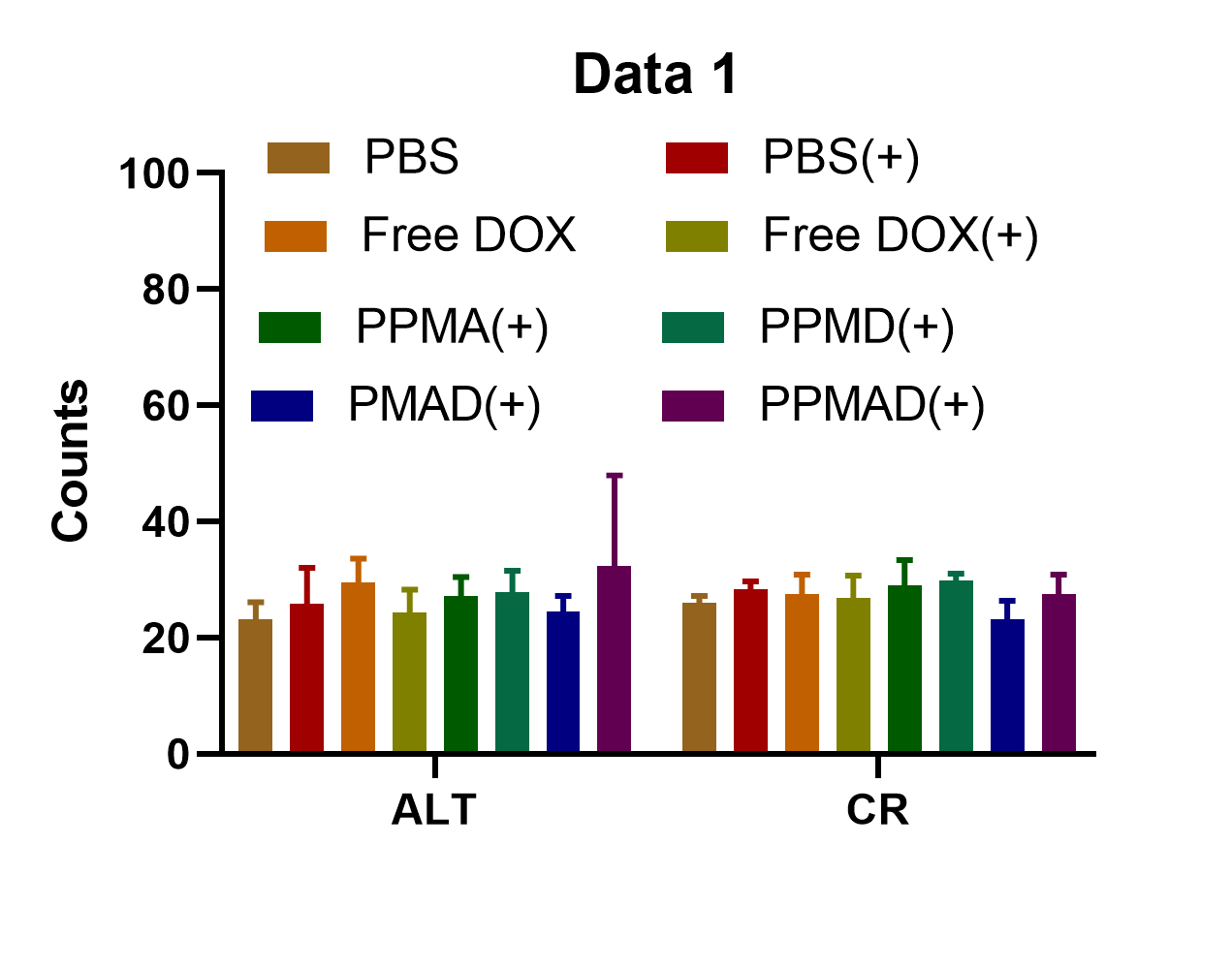


**Fig. S11** Blood biochemical analysis of mice after different treatment.
